# Two COWP-like cysteine rich proteins from *Eimeria nieschulzi* (coccidia, apicomplexa) are expressed during sporulation and involved in the sporocyst wall formation

**DOI:** 10.1186/s13071-015-0982-3

**Published:** 2015-07-25

**Authors:** Ernst Jonscher, Alexander Erdbeer, Marie Günther, Michael Kurth

**Affiliations:** Institute for Zoology, Technische Universität Dresden, 01062 Dresden, Germany

**Keywords:** *Eimeria*, OWP, *Cryptosporidium*, COWP, *Toxoplasma gondii*, TgOWP, Sporocyst wall, Oocyst, Circumplasm

## Abstract

**Background:**

The family of cysteine rich proteins of the oocyst wall (COWPs) originally described in *Cryptosporidium* can also be found in *Toxoplasma gondii* (TgOWPs) localised to the oocyst wall as well. Genome sequence analysis of *Eimeria* suggests that these proteins may also exist in this genus and led us to the assumption that these proteins may also play a role in oocyst wall formation.

**Methods:**

In this study, COWP-like encoding sequences had been identified in *Eimeria nieschulzi*. The predicted gene sequences were subsequently utilized in reporter gene assays to observe time of expression and localisation of the reporter protein *in vivo.*

**Results:**

Both investigated proteins, EnOWP2 and EnOWP6, were expressed during sporulation. The EnOWP2-promoter driven mCherry was found in the cytoplasm and the EnOWP2, respectively EnOWP6, fused to mCherry was initially observed in the extracytoplasmatic space between sporoblast and oocyst wall. This, so far unnamed compartment was designated as circumplasm. Later, the mCherry reporter co-localised with the sporocyst wall of the sporulated oocysts. This observation had been confirmed by confocal microscopy, excystation experiments and IFA. Transcript analysis revealed the intron-exon structure of these genes and confirmed the expression of EnOWP2 and EnOWP6 during sporogony.

**Conclusions:**

Our results allow us to assume a role, of both investigated EnOWP proteins, in the sporocyst wall formation of *E. nieschulzi*. Data mining and sequence comparisons to *T. gondii* and other *Eimeria* species allow us to hypothesise a conserved process within the coccidia. A role in oocyst wall formation had not been observed in *E. nieschulzi*.

**Electronic supplementary material:**

The online version of this article (doi:10.1186/s13071-015-0982-3) contains supplementary material, which is available to authorized users.

## Background

Coccidian parasites are known to form persistent stages, the oocysts, at the end of their life cycles. Within the oocyst, the sporozoites can either be formed directly without an additional protective structure, like in *Cryptosporidium*, or they are enclosed in a secondary cyst, the sporocyst, like it occurs *inter alia* among the genera *Eimeria*, *Isospora* or *Toxoplasma.*

The *Eimeria* oocyst wall consists of two layers originated by content of two different types of wall forming bodies [1]. Although antigens of one or both wall forming bodies (WFB) were characterized by different studies [2–4], but only the amino acid composition of proteins of WFBII, and thereby of the inner oocyst wall, could be elucidated and are known as GAM-proteins. These tyrosine rich GAM proteins are cross linked via dityrosines [3]. In *Cryptosporidium* cysteine rich proteins are known to be a part of the oocyst wall, designated as Cryptosporidium Oocyst Wall Protein; COWP [5]. Recently, in *Toxoplasma gondii* COWP homologous proteins had been identified, designated as TgOWP, and characterized to be related to the oocyst wall [6]. Additionally, there is also proteomic evidence for a role in sporocysts wall formation in *Toxoplasma gondii* [7]. COWP homologous sequences were also found and annotated in the genomes of several avian *Eimeria* species [8] but their function is unknown. It can be assumed that these COWP, respectively TgOWP homologous proteins from *Eimeria*, play a role in the wall formation process of the oocyst and/or sporocyst wall in *Eimeria*. To test this hypothesis we utilized the parasite *Eimeria nieschulzi* and the transfection technology to follow the expression and dynamics of two COWP-like proteins in *E. nieschulzi* (EnOWP) via reporter gene assays. The monoxenous life cycle of *Eimeria* results in difficulties generating transformed oocyst, which was conquered in the last years [9–12] but has the advantage to generate asexual and sexual stages in a single host. This is different to *Toxoplasma gondii*, where propagation and genetic modification of asexual stages *in vitro* is common, but experiments with cats, the definitive host of *T. gondii*, are subject to high requirements.

## Methods

### Identification of COWP homologous sequences in *Eimeria nieschulzi*

Genomic data of *Eimeria nieschulzi,* based on Illumina HiSeq 2000 (paired end) sequencing and *de novo* assembly with CLC Workbench 5.5 performed by Co. GATC Konstanz *(*GenBank ASM82694v1, M. Kurth, direct submission*)* were analyzed by TBLASTN search against TgOWP proteins sequences.

The identified contigs were analyzed concerning the existence of open reading frames encoding cysteine containing amino acid motifs. The protein e006Ecoding regions in the identified contigs were analyzed with SignalP 4.0. with respect to the occurrence of signal peptide containing open reading frames. The putative amino acid sequence in the last protein encoding exon was identified by similarity of the distance of cysteine encoding triplets and the occurrence of a stop codon in the same open reading frame, as well as the absence of putative splice donor sites. The identified, putative protein sequences were again analyzed by TBLASTN against predicted proteins in *Eimeria* species at GenBank and ToxoDB ([13] as well as in transcripts deposited at *Eimeria* Transcript database [14, 15]).

### Plasmids

The modified pDLY-T1 plasmid [12] pDLYmc with mCherry [16] was digested with *Hind*III and *Afl*II to remove the Etgam56 promoter and to replace it with the putative EnOWP promoter and/or promoter-gene-cassette.

**Fig. 1:**
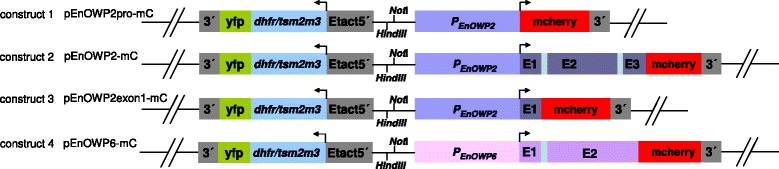
Schematic drawing of the arrangements and content of expression cassette. EnOWP reporter cassettes are head-to-head orientated to the chimeric selectable marker (dhfr/ts-yfp) Etact5′: *E. tenella* actin promoter, dhfr/tsm2m3: encodes a pyrimethamine resistant dihydrofolate reductase-thymidilate synthase [25], yfp: yellow fluorescent protein; 3′: 3′flanking region of *E.tenella* actin gene. Construct **1**: pEnOWP2pro-mC encodes the chimeric selectable marker and a mCherry gene under control of the EnOWP2-promoter; Construct **2**: pEnOWP2-mC encodes the chimeric selectable marker and a mCherry gene fused to the full length genomic EnOWP2 controlled by the authentic promoter. Construct **3**: pEnOWP2Exon1-mC encodes the chimeric selectable marker and a mCherry gene fused with the Exon1 of EnOWP2 controlled by the authentic promoter; Construct **4**: pEnOWP6-mC encodes the chimeric selectable marker and a mCherry gene fused to the full length genomic EnOWP6 controlled by the authentic promoter

The putative promoter-gene-cassette of EnOWP (named EnOWP6) in contig Enie_6026 (GenBank JRZD01006026) was amplified by PCR (KappaHifi, GC-Buffer, Co. Peqlab) using the primer combination #1 and #3. The PCR product was separated by agarose gel electrophoresis (1 % agarose, 0.5 % TBE-Buffer), purified from gel (GeneJet Gel Extraction Kit, Thermo Scientific) and cloned into pjet.1.2 plasmid using the CloneJet PCR cloning Kit (Thermo Scientific) according to the manufacturer’s recommendations. The isolated plasmid DNA of cultivated *E. coli* colonies was digested with *Bgl*II to control insert size and subsequently sequenced by GATC- Biotech (Konstanz, Germany) to confirm the insert sequence. Plasmids with the correct nucleotide sequence were digested with *Hind*III and *Afl*II and the purified fragment was cloned within the *Hind*III/*Afl*II sites of the pDLYmc plasmid. The resulting plasmid was named pEnOWP6-mC (construct 4) The putative promoter of EnOWP (named EnOWP2) in contig Enie_2812 (GenBank JRZD01002812) was amplified by primers #3 and #4, the entire promoter-gene-cassette by primers #3 and #5 as well as promoter-exon1-cassette by primer #3 and #6 and cloned respectively into pjet1.2 plasmid and later via *Not*I and *Afl*II in the pEnOWP6-mC plasmid, by replacing the EnOWP6 cassette. The plasmids were named pEnOWP2pro-mC (construct 1), pEnOWP2-mC (construct 2), and pEnOWP2exon1-mC (construct 3). Plasmids are schematically illustrated in Fig. 1. Suitable amounts (100–200 μg) of the plasmids for transfection experiment were produced with the peqGOLD XChange Plasmid Midi Kit (Peqlab). Plasmids were stored at −20 °C until use. (Primer sequences are listed in Additional file 1.)

### Parasites

Rats (*Rattus norvegicus*; Charles River Crl:CD(SD)) were orally inoculated by gavage with 500.000 sporulated oocysts of *E. nieschulzi.* Infected rats were anaesthetised by a mixture of oxygen and carbon dioxide (1:1) and finally euthanized with pure carbon dioxide at 168 h p.i. (+/− 1 h). The whole intestine was removed and disintegrated with a scissor to a mush like texture. The oocysts containing tissue mush was resuspended in a 2.5 % sodium dichromate solution (300 ml per intestine) and continuously agitated by a magnetic stirrer with magnetic stir bar about 3–5 days with permanent air supply. The oocysts were recovered by flotation with concentrated sucrose solution [17] and stored in 2.5 % potassium-dichromate at 4 °C. For the usage in transfection experiments, the sodium dichromate was removed by multiple washing with H_2_O, the oocyst containing pellet was resuspended in 3–4 ml pure sodium hypochlorite (12 % active chloride) and incubated about 30 min at room temperature. The sodium hypochlorite was removed by multiple washing with sterilised water and centrifugation.

Excystation and purification of sporozoites was performed as previously described [11, 18].

### Transfection and selection of transformed parasites

The transfer of plasmids into *E. nieschulzi* 20×10^6^ sporozoites was carried out by electroporation with approximately 100 μg *Not*I digested plasmid with 2 μl additional *Not*I enzyme for restriction enzyme mediated integration as previously described [11].

Infection of the rat and *in vivo* selection was performed as previously described [11, 12], with 300 mg/kg pyrimethamine for 4 d. To prevent contamination of the environment with genetically modified parasites, animals were kept in an individually ventilated cage system (Tecniplast) according to official regulations.

Rats were euthanized at 174 h p.i. (+/−1 h) and oocyst were isolated and further treated like wild type parasites as described above. Propagation of transformed oocysts was performed as described above, but with 5000 to 500.000 oocysts, depending on the oocyst output of the initial passage.

### Immunofluorescence assay

Sporocysts were excysted with pepsin and glass beads as previously described [18]. Sporocysts were prefixed with methanol at −20 °C for 10 min. After sedimentation, the sporocysts were diluted in 1×PBS (Phosphate Buffered Saline) and dropped on a object slide and dried at room temperature. An additional sample was dropped on the object slide without previous methanol treatment. Sporocysts were incubated with 5 % FBS (fetal bovine serum), 0.1 % Triton X-100 in 1×PBS, the untreated sample only with 5 % FBS in 1×PBS for about 60 min to block unspecific antigens. After 5 min washing with 1×PBS sporocysts were incubated with anti-mCherry antibody (Biorbyt, orb11618, produced in goat ) 1:200 in 5 % FBS, 0.1 % Triton X100 in 1×PBS about 120 min, a negative control without antibody and the untreated sporocysts without Triton x-100. After removal of the antibody solution and four times washing for about 5 min, sporocysts were incubated with secondary FITC (Fluorescein isothiocyanate) conjugated anti-goat antibody (SIGMA 1:200) in 5 % FBS, 0.1 % Triton in 1×PBS for about 60 min. After multiple washing (4×5 min) with 1×PBS, sporocysts were mounted on an object slide with Mowiol 4–88 (prepared according to the manufacturer description, Co. Carl Roth) and a cover glass. A control with wild type sporocysts was performed with and without MeOH/Triton treatment, and primary and secondary antibodies.

### Identification of EnOWP transcript sequences

Based on the observation of the reporter gene assay, sporulating oocysts (24 h) were superficially sterilised as described above. The oocyst pellet was mixed 1:1 with glass beads (diameter 0.5 mm) and strongly grinded on a vortex mixer for about 5 min. The successful disintegration was controlled by microscopy. RNA was extracted with the Roche High Pure RNA Isolation Kit. The glass beads/oocysts lysate was mixed with 400 μl 1xPBS 4 °C, then 800 μl binding buffer was added and mixed again. The supernatant was applied to the High Pure Filter Tube and we further followed the protocol supplied by the manufacturer. The cDNA synthesis was performed with the Thermo Scientific Maxima H Minus First strand cDNA Synthesis Kit according to the manufacturers instructions. The cDNA was purified from the PCR reaction assay with the Gelextraction Kit (Thermo Scientific) and eluted in ultrapure water. The transcripts were amplified with the primer pair #7 and #8 for COWP6 (Kappa Hifi polymerase (Peqlab), GC-Buffer) and with the primer pair #9 and #10 for COWP2 (Kappa Hifi polymerase (Peqlab), Fidelity buffer). The PCR-products with the expected size were purified from gel, cloned into pjet.1.2 (Clonejet, Thermo Scientific) and sequenced (Co. GATC, Konstanz). (Primer sequences are listed in Additional file 1).

### Transcript analysis

The stage specific expression of EnOWP was analysed by PCR using template cDNA from sporozoites, gametocytes and sporulating oocysts (24 h, see 2.6). Sporozoite cDNA was prepared from excysted sporozoites [18] and gametocyte cDNA from purified gametocytes (provided by Stefanie Wiedmer, unpublished) using the Roche High Pure RNA Isolation Kit in combination with the Roche cDNA synthesis Kit, according to the manufacturers recommendation. Controls were performed with the *E. nieschulzi* gam82 (GenBank AIN46826, primers #11 and #12) and ribosomal subunit [12]. (Primer sequences are listed in Additional file 1.)

### Identification of EnOWP2 and EnOWP6 orthologs in other Eimeria species

In the first instance we analyzed the translated mRNA sequence corresponding to contigs Enie_2812 (named EnOWP2) and Enie_6026 (named EnOWP6) via TBLAST against different databases (GenBank, Eimeria TranscriptDB, ToxoDB). Identified putative COWP2 and COWP6 encoding genomic sequences, respectively predicted cDNA sequences were aligned to the EnOWP2 and EnOWP6 cDNA and if applicable changed the predicted sequences (and in one case the genomic sequence) with the help of the intro-exon structure of the EnOWP-cDNAs.

### Microscopy

Micrographs were taken using Axiovert 135 with Colibri.2-light source (Zeiss), combined with the Olympus FviewII monochrome CCD-camera and the image processing software CellF Oylmpus and ImageJ. Excitation wavelength: 590 nm mCherry, 470 nm YFP and, 385 nm autofluorescence oocyst wall and Emission Filter Set HE62 (Zeiss).

The 3D-imaging of *Eimeria* oocysts was performed with an FV1000 confocal microscope (Olympus) equipped with a 100x/1.4 NA oil immersion lens, a motor stage, an Olympus Cellcubator with an epifluorescence lamp and differential interference contrast (DIC). We used a 488 nm laser and a 594 nm laser. The programme for imaging was FluoView (Olympus, Version 3.0) and the settings for EGFP or TexasRed were used respectively. The microscopy was carried out in uncoated ibiTreat 8-well μ-Slides by ibidi (Martinsried, Germany) at room temperature (22 °C). Therefore 200 to 300 μl of sporulating oocysts in 2.5 % potassium dichromate solution were transferred into one well of the slide and sealed with Parafilm. The oocysts were led to settle down for 10 min after they were transferred into the microscope. The epifluorescence lamp was used to find the parasites of interest manually. Then the oocysts were imaged with the following parameters: laser intensity 7–10 %, dwell time 8–18 μs, stack step size 0.37 μs, zoom level 0–3. Several times a 2-3x Kalman mode was used additionally. The collected stacks were then converted into 3D-reconstruction with the Imaris package (Bitplane, Version X64.7.1.1).

## Results

### Identification of COWP orthologs in *Eimeria nieschulzi*

By BLAST analysis we were able to identify contigs (1113, 6026, 2812) encoding putative COWP homologous proteins in *E. nieschulzi.* In contig Enie_2812 and contig Enie_6026 we found open reading frames with more than 1 kb reading encoding amino acid sequences with cysteine motif as described *in Cryptosporidium parvum* [5] and *Toxoplasma gondii* [6]. Homologous COWP encoding sequences to Enie_contig2812 and Enie_Contig6026 had been already annotated as putative COWP sequences in *Eimeria sp.* [8]. Upstream a large virtual open reading frame, a shorter virtual open reading frame encoding a putative signal peptide was found in each case, supposed to be the start of the protein encoding sequence. These gene predictions were used to design reporter plasmids with genomic sequences to observe the expression in transformed parasites (Fig. 1). The exact intron-exon borders had been identified by sequencing of cDNA.

### Sequence analysis of EnOWP2 and EnOWP6

To analyze the COWP homolog initially found in the *Eimeria nieschulzi* genomic contigs Enie_2812 (GenBank Accession JRZD01019969) and Enie_6026 we made BLAST analysis of translated sequences, of the obtained cDNA, against *Toxoplasma gondii* proteins. Using blastp at Toxodb.org the lowest E-value and therewith the highest similarity was found between EnOWP corresponding to contig Enie_2812 and TgOWP2, as well as EnOWP from Contig Enie_6026 and TgOWP6. For that reason EnOWP (Enie_contig2812) was named EnOWP2, respectively EnOWP6 (Enie_Contig6026). For complete results see in Additional file 2, paragraph C. The EnOWP2 gene has a length of 1511 bp (from start to stop codon) and harbours two introns of 53 and 54 bp length respectively.

**Fig. 2:**
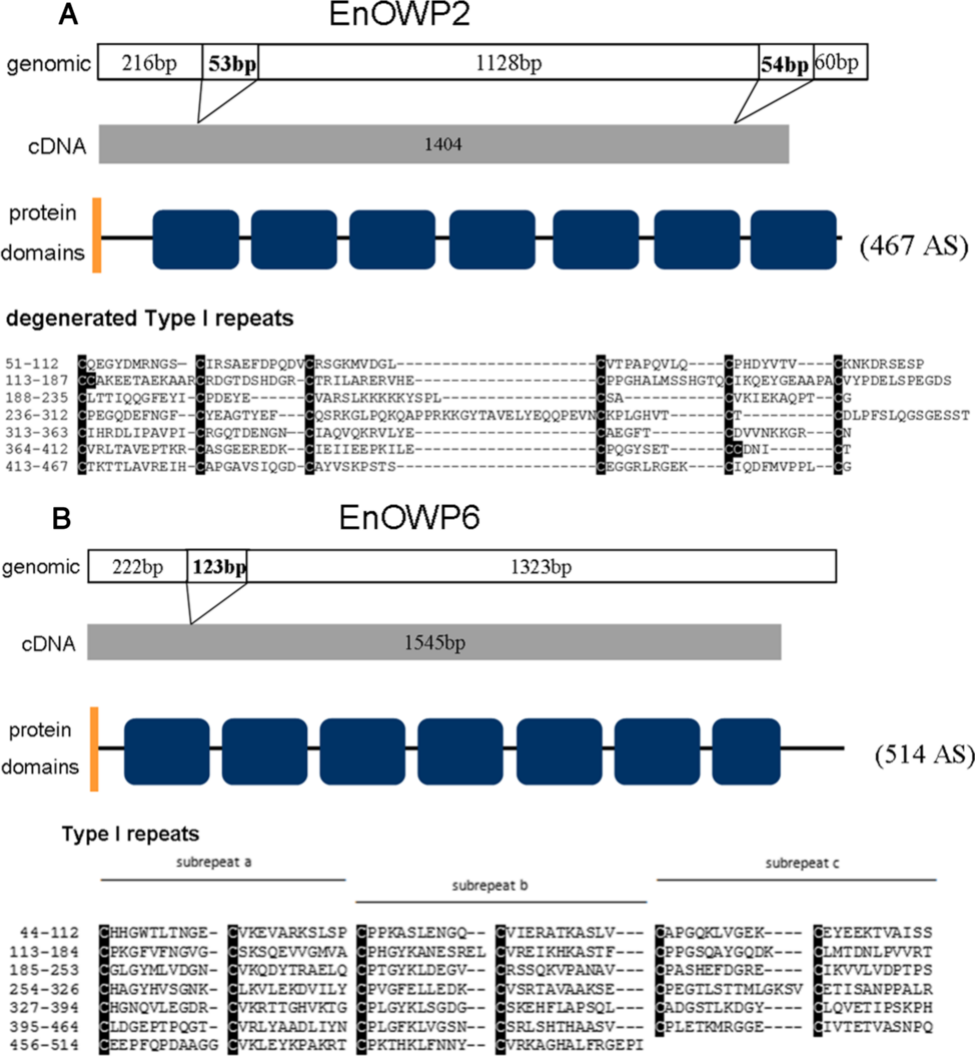
Structure of EnOWP2 (**a**) and EnOWP6 (**b**). **a**: The *E. nieschulzi* EnOWP2 gene has a length 1511 bp from the start to the stop codon. It harbours two intron of 53 bp and 54 bp length. The transcripts encodes 467 amino acids within 1404 bp. Cysteine residues occur in so called degenerated type I repeats, each repeat contains six cysteine residues, but in irregular distances. **b**: The *E. nieschulzi* EnOWP6 gene has a length 1660 bp from the start-to the stop codon. It harbours one intron of 123 bp length. The transcripts encode 514 amino acids within 1537 bp. Cysteine residues occur in type I repeats, each repeat contains six cysteine residues in regular distances similar to TgOWP6

The EnOWP6 mRNA encoded for 467 amino acids with cysteine residues in varying intervals (Fig. 2) comparable with TgOWP6 (see Additional file 2 paragraph G). The EnOWP6 gene has a length (from start to stop codon) of 1660 bp and harbours an intron of 123 bp length resulting in mature mRNA of 1537 bp which encodes for 514 amino acids. The transcript of both proteins were amplified from cDNA of sporulating oocysts but were not able to get amplified from gametocyte or sporozoite stage derived cDNA (Additional file 3).

### EnOWP2-promoter controlled mCherry expression

**Fig. 3:**
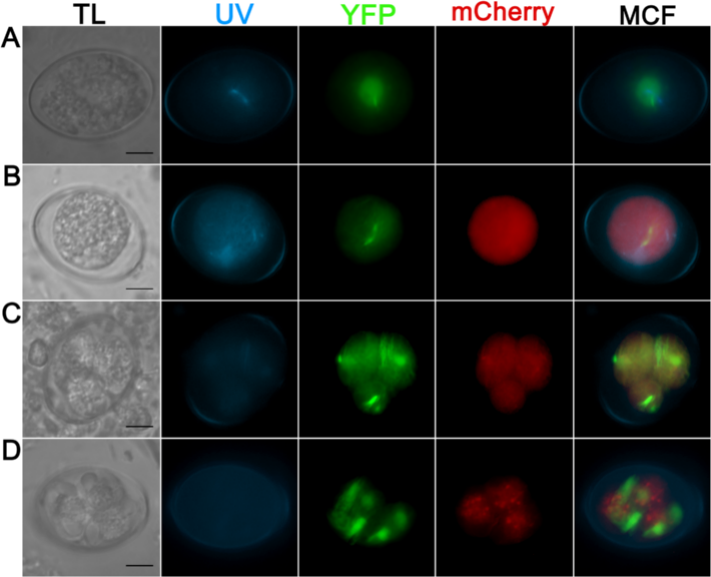
Transgenic E*. nieschulzi* oocyst expressing DHFR/TS-YFP (green) and EnOWP2-promoter controlled mCherry (red); construct 1. **a**: Freshly harvested unsporulated oocyst 0.5 h post isolation with nuclear and cytoplasmatic YFP fluorescence signal. **b**: Sporulating oocyst at 19 h post isolation with YFP and mCherry signals in the condensed cytoplasm. **c**: Sporulating oocyst at 33 h post isolation showing the mitotic division in the formed sporoblasts. YFP and mCherry are localised to the cytoplasm. **d**: Sporulated oocyst at 3 d post isolation. YFP signal are localised to cytoplasm and nucleus of the sporozoites, mCherry signals are localised to sporozoite cytoplasm and the sporocyst residue (granular signal). Scale bar: 5 μm. **TL**: transmitted light; **UV**: UV auto fluorescence of the oocyst wall; **YFP**: YFP fluorescence; mCherry: mCherry fluorescence; **MCF**: multichannel fluorescence, overlay of all fluorescent signals in the row

The mCherry-protein under control of a ca. 1.6. kb putative promoter (pEnOWP2pro-mC, construct 1) was not detected in freshly harvested oocyst after the initial or continuing passage. The yellow fluorescent protein of the selectable marker cassette was observed in the cytoplasm of the oocyst. In these, visibly transgenic oocysts, the red fluorescent mCherry signal became visible after 12 h in the oocysts cytoplasm and remained there during the sporoblast formation. Finally, it was detectable in the cytoplasm of sporozoites and the sporocyst residuum in the sporulated sporocyst (time course and signal pattern shown in Fig. 3). The mCherry signals were constantly present in all sporocysts of transgenic oocysts, while the YFP signals changed in the sporulated oocysts. The phenomenon was investigated in a second passage of the pEnOWP2pro-mC-oocysts. This resulted in 50 % transgenic oocysts (sporulated) with both signals, YFP and mCherry (randomly counted sample n = 52 oocysts). 46 % (12) of these oocysts expressed the YFP in all four sporocysts, 35 % (9) in two sporocysts, 15 % (4) in one and 4 % in three (1). Also in this passage, the mCherry signal was constantly distributed to all four sporocysts if expressed (see Additional files 4, 5 and 6).

### Localisation of EnOWP2-mCherry reporter signals during sporulation

In a second reporter gene assay, the fusion of the putative EnOWP2 full-length gene with mCherry under control of the authentic promoter (construct 2) the mCherry signal was also absent in freshly harvested oocysts. After 12 h of sporulation, mCherry was observed extracytoplasmatically between the oocyst cytoplasm and the oocyst wall, coincident with the condensation of the cytoplasm. We designated this, so far unnamed compartment, as ‘circumplasm’.

**Fig. 4:**
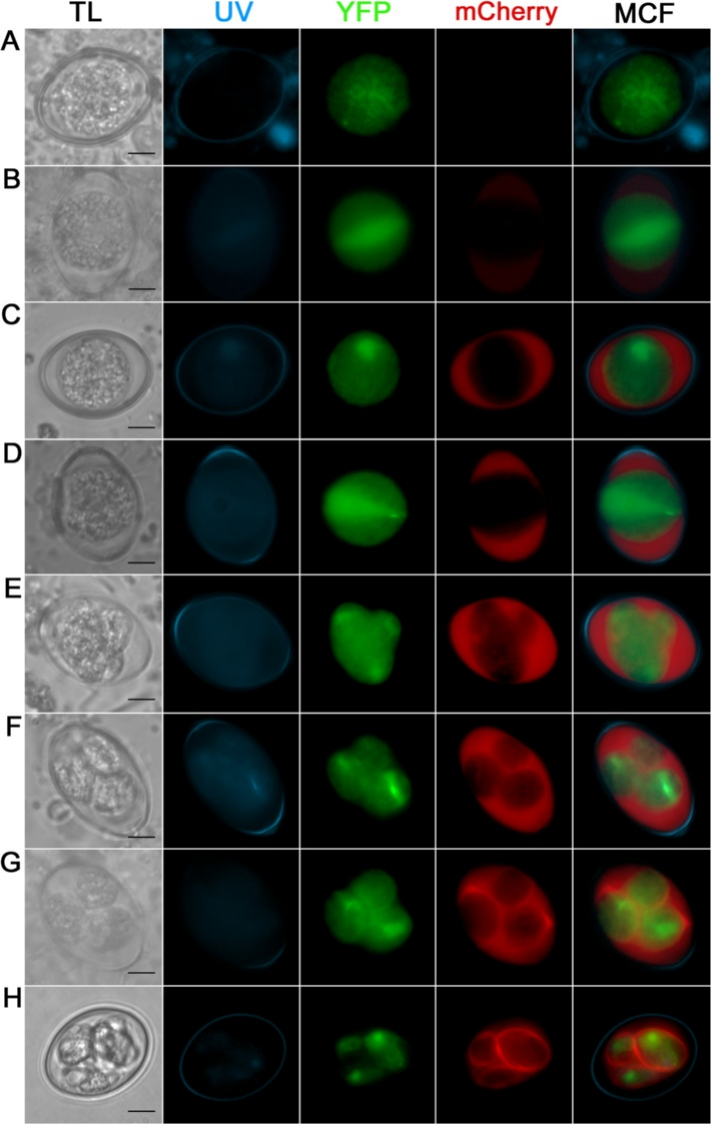
Expression pattern of the EnOWP2-mCherry-reporter (construct 2) during oocyst sporulation. **a**: Freshly harvested oocyst with YFP but absent mCherry signal. **b**: Oocyst after 12 h sporulation with cytoplasmatic and nuclear YFP signal and weak extracytoplasmatic mCherry signal in the structureless space between the condensed cytoplasm and the oocyst wall, designated as ‘circumplasm’. **c**: Oocyst after 17 h sporulation with strong mCherry signal in the circumplasm. **d**: Oocyst after 18 h during first meiotic reduction division. **e**: Sporulating oocyst during sporoblast formation. **f**: Sporulating oocyst after sporoblast formation at 24 h sporulation. **g**: Sporulating oocyst after. 26 h sporulation with mCherry signals in the circumplasm and protein accumulation at one end of the sporocyst. **h**: Full sporulated oocyst after 4 d sporulation. The mCherry signal surround the YFP signal and co-localise apparently with the sporocyst wall. Scale bar 5 μm. **TL**: transmitted light; **UV**: UV autofluorescence of the oocyst wall; **YFP**: YFP fluorescence; **mCherry**: mCherry fluorescence; **MCF** (multichannel fluorescence) overlay of all fluorescent signals in the row

The mCherry signal remained in this circumplasm during meiotic division and formation of sporoblasts and later became condensed at one end of the sporocysts. Within two hours, the mCherry signal was co-localised with the sporocyst wall and in some cases also little in the sporocyst residuum (pattern and time course of expression shown in Fig. 4). The mCherry signals in the circumplasm were initially still present, but much less intense compared to the sporocyst wall. The mCherry maintained in the sporocyst wall during the complete observation period for about ten months without any changes. YFP signals were observed in the sporozoites for the same period. In the initial passage (with transfected sporozoites, named P0) we got 33 % fluorescent oocyst. From the repeated passage, of the initially received oocysts, we got 42 % transgenic oocysts (P1). From this passage (P1) 96 % of the transgenic oocysts were expressing mCherry, and YFP with a different distribution of the YFP signal in the sporocysts (see Table 1). However, the mCherry signal was visible at the wall of all four sporocysts if YFP was visibly expressed, independently from the number of YFP expressing sporocysts within a single oocyst (for phenotypes see Additional file 4: Figure S1). In a further passage (P2) the proportion of transformed oocysts (at least one fluorochrome was expressed) was increased up to 56 %. Out of these fluorescent oocysts, 100 % showed mCherry in each sporocysts wall and YFP in at least one of the sporocysts. The YFP signals within these mCherry positive oocysts showed similar distribution of YFP to the sporocysts like the previous passage. In a further passage we could increase the proportion of transformed oocysts up to 64 %. All counts are summarised in Table 1.

**Table 1:** Proportion of transgenic oocysts (at least one fluorochrome is visibly expressed) in the single passages of parasites transformed with the pDLY-EnOWP2-promoter_mC, pDLY_EnOWP2_mC, pDLY_EnOWP2Exon1_mC, and pDLY_EnOWP6_mC construct and selected by pyrimethamine

Construct	P	Part of transformed oocysts^a^ (trOoc/total)	Part of mcherry + oocysts in trOoc*	Part of yfp + oocysts in trOoc	Proportion of YFP expressing sporocyst per transformed sporulated oocyst^b^			
					1	2	3	4
*construct 1*	P0	n.d.	n.d.	n.d.	n.d	n.d.	n.d.	n.d.
EnOWP2 promoter mCherry	P1	50 % (26/52)	100 % (26)	100 % (26)	15 %	35 %	4 %	46 %
*construct 2*	P0	33 % (41/123)	95 % (39)	100 % (29)	12 %	24 %	7 %	56 %
EnOWP2-mCherry	P1	42 % (75/180)	96 % (72)	100 % (75)	11 %	37 %	5 %	47 %
	P2	56 % (74/131)	100 % (74)	100 % (74)	16 %	33 %	5 %	45 %
	P3	64 %(102/159)	100 % (102)	97 % (99)	20 %	27 %	6 %	43 %
*construct 3*	P0	n.d.	n.d.	n.d.	n.d	n.d.	n.d.	n.d.
EnOWP2-Exon1_ mCherry								
	P1	26 % (45/170)	67 % (30)	100 % (45)	4 %	40 %	4 %	51 %
*construct 4*	P0	36 % (45/123)	42 % (high)	100 % (45)	9 %	29 %	7 %	55 %
EnOWP6 -mCherry (construct 4								
	P1	21 % (25/120)	96 % (24) (46 % high 54 % low)	100 % (25)	16 % (4) mC low	16 % (4) mC low	8 % (2) mC low	60 % (15) mC high: 11 mC low: 3 no mC: 1)

^a^at least one fluorochrome microscopically visible *

^b^rounded, n.d. not determined

### Excystation of EnOWP2-mCherry sporocysts and sporozoites

**Fig. 5:**
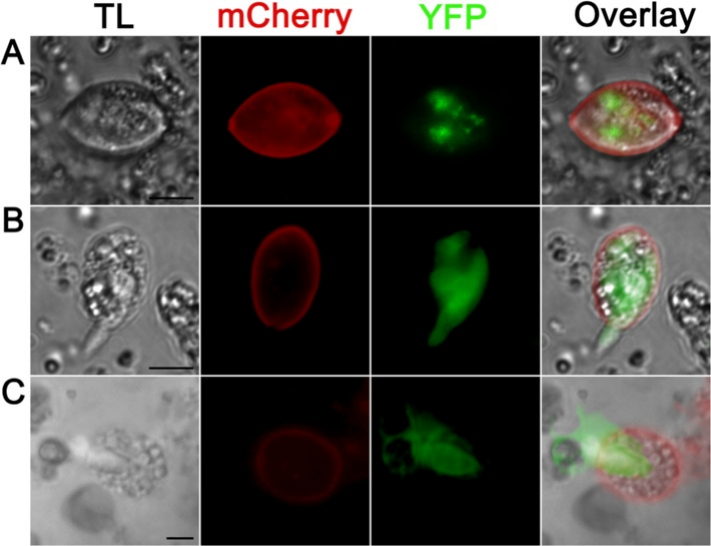
Excystation of sporocyst and sporozoites from pEnOWP2_mCherry transformed oocysts **a**: Free sporocyst **b**: Sporocyst during sporozoite excystation. The first of two YFP expressing sporozoites had started to leave the sporocyst **c**: Sporocyst during sporozoite excystation. The second (of two sporozoites within a sporocyst) is leaving a sporocyst. The red fluorescent sporocyst wall and non fluorescent material remains autonomous from the green fluorescent signals of sporozoite. Scale bar 5 μm. **TL**: transmitted light; **YFP**: YFP fluorescence; **mCherry**: mCherry fluorescence; **MCF** (multichannel fluorescence) overlay of all fluorescent signals in the row

To investigate if EnOWP2-mCherry fusion protein is associated with the sporocyst wall we investigated the fluorescence signal by confocal microscopy, during the excystation process and after immunofluorescence test with an anti-mCherry antibody. Sporocysts were excysted by pepsin and glass beads examined under the microscope before and during the incubation with excystation medium. Initially we observed free sporocysts with and without YFP signals. The mCherry signal was concentrated and co-localised to the sporocyst wall and the polar regions of the sporocyst. Induced by the excystation medium we observed the exit of sporozoites through the polar region of the sporocyst with absent mCherry signals (see Fig. 5). The majority of the red mCherry signals were located at the sporocyst wall, during the sporozoites showed only the YFP signals. After excystation empty sporocysts with mCherry fluorescent wall was left and non fluorescent residual material was left. The mCherry fluorescent signals of the sporocyst walls were not stable during these conditions and disappeared within 15–30 min and only YFP signals of the sporozoites were visible in the excystation assay.

### Immunofluorescence assay with anti-mCherry for EnOWP2mc sporocysts

**Fig. 6:**
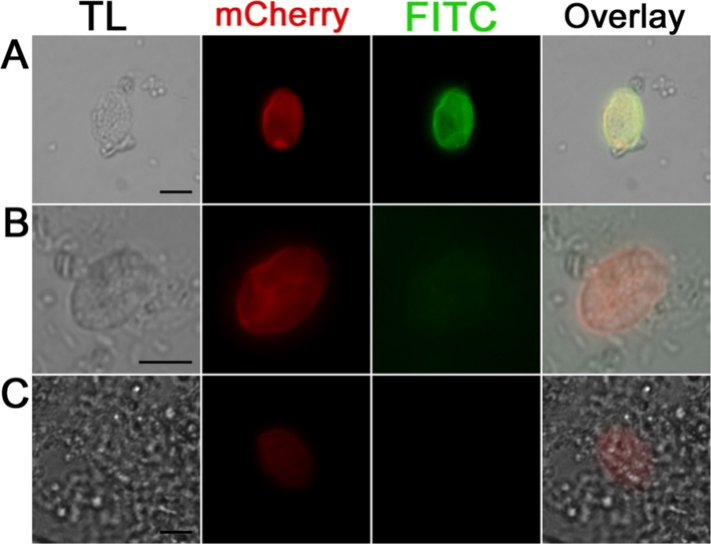
Immunofluorescence detection of mCherry in the sporocyst wall of mCherry positive but YFP negative sporocysts with goat anti-mCherry antibody and secondary anti-goat FITC antibody. **a**: Methanol fixed and Triton treated sporocyst incubated with primary anti-mCherry antibody and secondary anti-goat FITC antibody shows the signal of mCherry and the FITC signal of the antibody interaction with the sporocyst. **b**: Same antibody combination like in **a** but without Methanol fixation and Triton treatment shows similar mCherry signal, but a much reduced FITC signal. **c**: Methanol fixed sporocyst with Triton X100 treatment, without primary (anti mCherry) antibody but with secondary anti-goat FITC antibody does not show green fluorescent signal (K). Scale bar 5 μm. **TL**: Transmitted light micrograph, **mCherry**: mCherry signal of the sporocyst **FITC**: Signal of the anti-Goat FITC Antibody

To confirm localisation of mCherry in sporocyst walls of EnOWP2mc parasites, we incubated sporocysts with primary goat anti-mCherry and FITC conjugated anti-goat antibodies. Due to the fact of the stability and visibility of the fluorochrome after methanol or the drying process, we evaluated this test only on the sporocyst phenotype with present mCherry-signals but without endogenous YFP positive sporozoites. On methanol fixed and Triton X-100 treated sporocysts we observed red (stable mCherry reporter) and green fluorescent (antibody based) sporocysts, with stronger signals at the sporocyst wall (Fig. 6). Without secondary antibody no green signals were observed. However red fluorescent mCherry signals were still present. On just dried sporocysts we observed only a weak green fluorescent signal but same red fluorescence. On wild type sporocysts neither red fluorescent nor green fluorescent signals were detectable independently of the application of methanol and triton x-100 treated or drying. (Additional file 7: Figure S3).

### Confocal microscopy of EnOWP2-mCherry oocysts

**Fig. 7:**
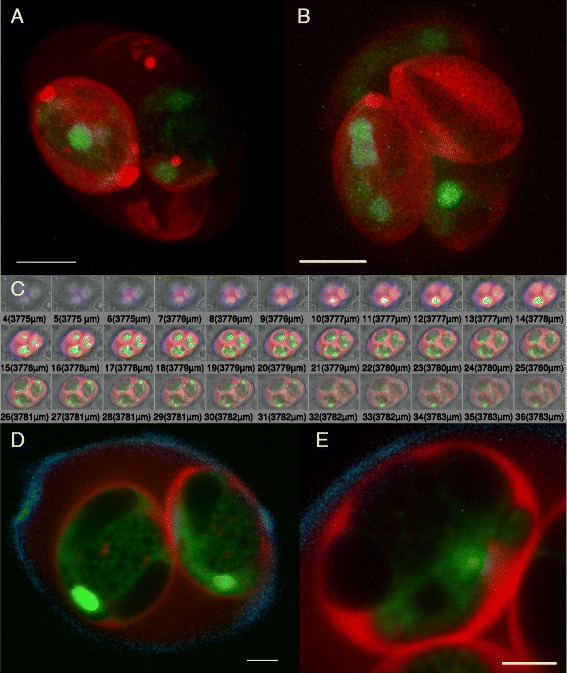
Confocal Microscopy of sporulated EnOWP2-mCherry-oocyst. **a**: 3D reconstruction of optical sections through an EnOWP2-mCherry oocyst (red and green channel). The mCherry signals are distributed at the sporocyst wall around the cytoplasmatic YFP signal. More intense signals are localised at both polar ends of the sporocyst. Note, that all sporocysts harbour the red mCherry signal, whereas YFP is only expressed within two sporocysts (see also Additional file 5). Scale bar 5 μm. **b**: oocyst with mCherry signal in all sporocyst but YFP in 3 sporocysts. More intense mCherry signals are visible at the Stieda body region (see also Additional file 6) Scale bar 5 μm. **c**: Selection of optical sections of representing fluorescence signal through the half of an oocyst. **d**: Optical section through the middle of an oocyst (overlay of mCherry, YFP and UV autofluorescence). The mCherry signals are localised at the sporocyst wall, few vesicle shaped signals in the sporocyst residuum and also less mCherry signals in the circumplasm (see also Additional file 9) Scale bar 2 μm. The YFP is distributed to the sporozoites within the sporocysts. **e**. Optical section through an oocyst and close up of a single sporocyst (overlay of mCherry, YFP and UV autofluorescence). Strong mCherry signals separating the sporocyst from the circumplasm. Within the sporocyst the red mCherry signals embed the sporozoites, indicated by the green YFP signals. Scale bar 1.5 μm

By confocal microscopy it was possible to get higher resolution concerning the distribution of COWP2 protein via its mCherry reporter signal in pDLY_EnOWP2mc transformed oocyst (construct2). The mCherry reporter was found in transgenic oocyst limiting the sporocyst at the outside (Fig. 7a and b). It was observed in all sporocyst walls of transgenic oocysts, independently from the presence of YFP signals in the sporocysts of the oocyst. More intensive mCherry was observed at the polar regions of the sporocyst. Optical sections (Fig. 7c) revealed a distinct limitation of mCherry signal in direction to the circumplasm but not on the inside, where the sporozoites appeared to be embedded in the mCherry reporter protein (Fig. 7d and e).

### Localisation of truncated EnOWP2Exon1-mCherry reporter signals

The time of expression of the EnOWP2Exon1-mCherry (construct 3) was comparable to that of construct 1 and 2. In freshly harvested oocysts no mCherry expression was observed. After a sporulation period of 12 h, the mCherry signals became visible in the circumplasm. We observed also vesicle like signal at the boundary of the cytoplasm, which were later distributed more centrally in the cytoplasm. Within sporoblast formation, the mCherry disappeared from the circumplasm and was observed within the sporoblast surrounding the cytoplasmatic YFP signals. The mCherry appeared to be associated with the sporocyst wall, but seems not to be accumulating, as it has been observed with the full length protein.

**Fig. 8:**
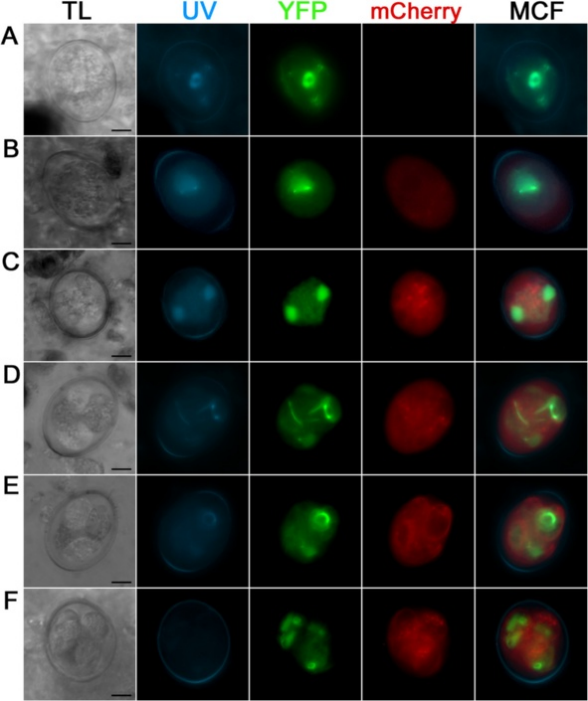
Fluorescence pattern of EnOWP2Exon1-mCherry (construct 3). **a**: Freshly harvested oocyst with cytoplasmatic YFP but absent mCherry signal. **b**: Oocyst after 16 h sporulation with cytoplasmatic YFP signal and weak intra- and extracytoplasmatic mCherry signal. Red fluorescent vesicle-like signals are visible at the border of the cytoplasm. **c**: oocyst at the end of first meiotic division at 26 h sporulation **d**: oocyst during sporoblast formation with cytoplasmatic and nuclear associated YFP signal, as well as extracytoplasmatic mCherry signal, vesicle-like mCherry signal are in the sporoblast are visible as well ; 26 h sporulation period. **e**: oocyst with sporoblast during sporozoite formation after 36 h sporulation period. The mCherry signals disappear from the extracytoplasmatic space and appear associated with the sporocyst wall. **f**: Fully sporulated oocyst after approx. 2d sporulation period. The mCherry signal surround the YFP signals close to the sporocyst wall and are also localised to the sporocyst residuum (granular signal). Scale bar 5 μm. **TL**: transmitted light; **YFP**: YFP fluorescence; **mCherry**: mCherry fluorescence; **MCF** (multichannel fluorescence) overlay of all fluorescent signals in the row

In fully sporulated oocysts the mCherry was found in the vesicle like bodies and in the surrounding area of the sporozoites within the sporocyst. It appeared to be associated with the sporocyst wall but not in the same intensity like in the EnOWP2-mC or EnOWP6-mC construct transformed *E. nieschulzi* oocysts (Fig. 8).

### Localisation of EnOWP6-mCherry reporter signals and excystation

After the initial infection with transfected sporozoites (construct 4) and isolation of oocysts from the host animal we found wild type oocysts, YFP expressing oocyst and a few oocysts with YFP and mCherry signals as well. However, macrogamonts showed no mCherry signal (Additional file 8: Figure S4). The red fluorescence was found at the border of the cytoplasm, apparently associated with the inner oocyst wall. After a few hours, when the cytoplasm became contracted, mCherry was found in the circumplasm. A co-localisation with the inner oocyst wall was not confirmed.

**Fig. 9:**
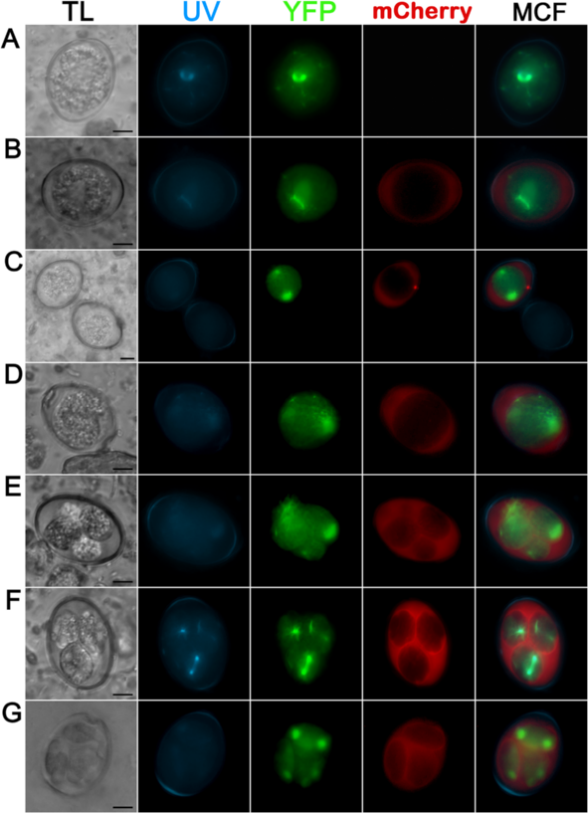
Expression pattern of the EnOWP6-mCherry reporter during sporulation (construct 4) Bar 5 μm. **a**: Freshly harvested oocyst with cytoplasmatic YFP but absent mCherry signal. **b**: Oocyst after 6 h sporulation period with extracytoplasmatic signal and cytoplasmatic YFP signals. **c**: Comparison between transgenic oocyst with mCherry and YFP signal and wild type oocyst with only UV autofluorescence after 19 h sporulation period. Transgenic oocyst with cytoplasmatic and nuclear YFP signal, indicating first meiotic division, and extracytoplasmatic mCherry signals as well as a punctuated mCherry signals in the circumplasm. **d**: Transgenic oocyst after 20 h sporulation period with extracytoplasmatic mCherry and cytoplasmatic and nuclear YFP signal during first meiotic division. **e**: Oocyst after sporoblast formation (27 h sporulation period), the sporoblast with YFP signals is surrounded by extracytoplasmatic mCherry signals. **f**: Oocyst during sporozoite and sporocyst formation. The mCherry signals accumulate at the boundary of the sporoblast, probably involved in the formation emerging sporocyst wall, nuclear YFP signal within the sporoblast are indicating a mitotic process. Sporulation period 27 h **g**: Sporulated oocyst after 2d sporulation period. The red mCherry signals are mainly distributed to the sporocyst wall and are reduced in the circumplasm. Scale bar 5 μm. TL: transmitted light; YFP: YFP fluorescence; mCherry: mCherry fluorescence; MCF (multichannel fluorescence) overlay of all fluorescent signals in the row

**Fig. 10:**
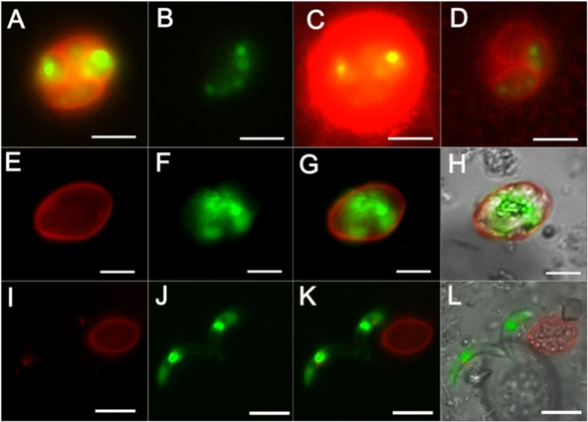
Varying mCherry signal intensity in EnOWP6-mCherry oocysts and excystation. **a** and **b** show two oocysts in the same visual field and 10s exposure signal in the red mCherry channel. A shows strong mCherry signal and YFP expression in all four sporocysts. **b**: shows no mCherry expression and YFP expression in two sporocysts. **c** and **d**: Images from **a** and **b** after image processing (intensity, saturation). The signals from A are clearly overregulated (**c**) but hidden signal in **b** became visible in **d**. The EnOWP6_mCherry was detected in nearly all investigated oocysts (see also Table 1) and always in four sporocyst per oocyst whereas the YFP signals altered. **E**-**L**: Excystation of sporocysts and sporozoites from EnOWP6-mCherry oocysts. **E**-**H**: Free sporocyst. The mCherry signals clearly margin the sporocyst and surround the green fluorescent signal of the sporozoites. **I**-**K**: Recently, excysted sporozoites (green) next to its abandoned sporocyst with non fluorescent material inside and a red fluorescent sporocyst wall. **A**-**D**: Overlay of mCherry and YFP fluorescence, Scale bar 10 μm.** E** and **I**: mCherry; F and **J**: YFP; G and K: overlay of mCherry an YFP; **H** and **L**: overlay of mCherry, YFP, and bright field images. **E**-**H**: Scale bar 5 μm; **I**-**L**: Scale bar 10 μm

The previous observation was probably caused by a combination of early expression of EnOWP6-mC, limited space in the emerging circumplasmatic space and bleed-through effects of the autofluorescent properties of the inner oocyst wall. The mCherry signals remained in the circumplasm until the four sporoblasts were formed. During sporozoite formation the red fluorescence became all-over concentrated at the sporoblast boundary, without any obvious concentrations at the region of the Stieda body. Micrographs of the single fluorescence signals are shown in Fig. 9. In fully sporulated oocyst, mCherry was predominantly found in the sporocyst wall and also in the circumplasm. In the initial passage we determined 36 % transgenic oocysts (YFP positive) but only a part of the fully sporulated oocysts visibly expressed YFP and mCherry (42 %). After prolonged exposure time and image processing we could visualise a signal in the sporocyst walls of this apparently mCherry negative sporocysts. This weak signal occurred in oocysts with YFP signals only in one to three sporocyst. In the most oocysts with four YFP positive sporocysts we found a strong visible signal. However low expression was also possible in this phenotype (see Table 1). Generally, the number of transformed oocysts was decreased in the passage P1 compared to P0 in contradiction to EnOWP2, where increasing of transformed oocysts with every passage was determined. Observations made on excysted sporocyst or during the excystation of sporozoites (Fig. 10) were comparable to EnOWP2-mCherry. Solely, accumulation of mCherry signals at the Stieda body region of the EnOWP6-mCherry sporocysts was not observed.

### Sequence comparison of EnOWPs with other Coccidia

By BLAST we could identify corresponding sequences (homolog to EnOWP2 and EnOWP6) in the Eimeria Transcript Database (Eimeria TDB) and the deposited data on ToxoDB. In comparison of both protein sequences with *Toxoplasma gondii* data we got the highest similarity with TgOWP2 and TGOWP6 (see above). EnOWP2 and TgOWP2 (TGVEG_209610) have a similar intron-exon structure (53/54 bp vs. 55/57 bp), nearly the identical number of encoded amino acids (467 vs. 462) and time point of expression in both species (sporulating oocyst). Amino acid sequences display the same degenerated type I repeats with similar distances of the cysteine residues. In comparison of EnOWP2 with sequences data set to *Eimeria* species we found corresponding EST in *E. tenella*, *E. acervulina* and *E. maxima* in the Eimeria transcript database, as well as at ToxoDB (see Additional file 2 paragraph E).

The stage specific expression data of three avian Eimeria species (Eimeria TDB Sequence IDs: Emax_264, Eten_1237, Eace_0293) confirm expression of Eimeria OWP2 in sporulating oocysts.

**Fig. 11:**
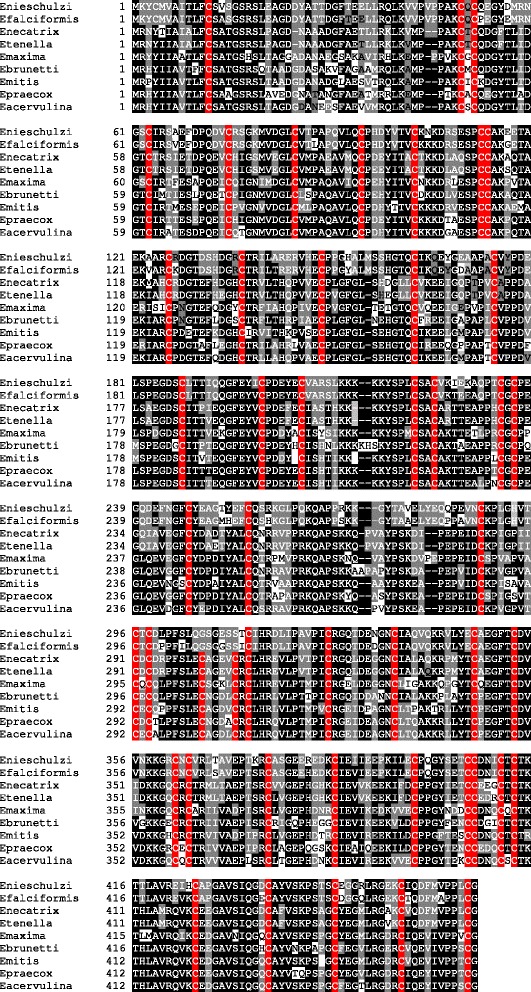
Multiple alignment of OWP2 orthologs The multiple Alignment of Eimeria OWPs sequences from avian and rodent *Eimeria* species (see Additional file 2 paragraph A) shows high similarity and conservation of this protein within the investigated *Eimeria* species. Cysteine residues are highlighted with a red background. Dominant amino acids (or gaps) with a proportion of more than 50% at the same position within the aligned sequences are highlighted with the black background colour. If at least two sequences share an amino acid (or gap) at the same position we highlighted this with a grey background colour. Amino acids which are unique at the particular position have a white background colour

The ToxoDB Gene IDs of *Eimeria acervulina* (EAH_00033530) *Eimeria maxima* (EMWEY_00029600) were in accord with our data. The corresponding protein predictions for *Eimeria tenella* (ETH_00025735), *Eimeria necatrix* (ENH_00081330), *Eimeria brunetti* EBH_0046800, *Eimeria mitis* (EMH_0001380), *Eimeria praecox* (EPH_0005040) and as well as *Eimeria falciformis* (EfaB_PLUS_2387.g293) showed high similarity, but the second intron was often disregarded. We manually reinvestigated *in silico* the genomic DNA sequences of these *Eimeria* species and could find splicing donor and acceptor sites at conserved positions (Additional file 2 paragraph B). The transcript predictions were changed accordingly to these findings (Additional file 2 paragraph A). In the case of *Eimeria praecox*, two assembly gaps were present in the locus of the COWP2 ortholog. These gaps were flanked by identical, probably duplicated sequences and we assumed an initial misassembly of the genomic sequences. We recovered this manually on the base of the conserved gene sequences of the other *Eimeria* species (see Additional file 2 paragraph A). Alignment of EnOWP2 with all existing and new predicted protein sequences shows high conservation of amino acid among all investigated *Eimeria* species (Fig. 11). EnOWP6 orthologs were found in expressed sequence tags (EST) of *E. acervulina* and *E. maxima* and *T. gondii* (Additional file 2 paragraph E). Compared to *T. gondii* it possesses the highest similarity with (see Additional file 2 paragraph C) which is also expressed in the sporulating oocyst and has a similar intron-exon structure and type I repeats (ToxoDB Gene-ID TGVEG_286250). EnOWP6 is highly conserved with predicted gene of other *Eimeria* species. The highest similarity was found with *Eimeria falciformis* (EfaB_MINUS_1001.g115), but also with *E. tenella* (ToxoDB Gene-ID ETH_00012470) and *E. necatrix* (ENH_00025410) high similarities were evident. The cysteine residues had almost the same distances in all EnOWP6 orthologs, but those from avian *Eimeria* sp. had an additional cysteine residue (Additional file 2 paragraph H) and in the N-terminal region, within the predicted signal peptide, and in the middle of the protein, poly alanine stretches were present (Additional file 2 paragraph H). Stage specific expression data at Eimeria TDB indicate, that expression of EnOWP6 orthologs from *E. acervulina* (Eimeria TDB Sequence ID Eace_0779) and *E. maxima* (Eimeria TDB Sequence ID Emax_0113) is upregulated in unsporulated oocysts, but not in sporulating oocysts (see Additional file 2 paragraph E).

## Discussion

A *Cryptosporidium* oocyst wall protein, so called COWP, was originally identified and characterized from *C. parvum* [5]. COWP belongs to a multigene family of nine different proteins in *Cryptosporidium* (COWP1-COWP9) [19] and a homolog in *Toxoplasma gondii* (TgOWP1) could be found. COWPs contain motifs (type I and type II) with cysteine residues in conserved and regularly spaced positions [5]. The cysteine residues, so assumed, form disulfide bridges among each other and are responsible for the stabilization and formation of the oocyst wall. Two different types, type I and type II repeats, had been described [5]. Both types of repeats contain six cysteine residues. Type II repeats are shorter than type I repeats and alternated by histidine residues of variable length. The *T. gondii* homolog TgOWP1 consists only of type I repeats and lacks type II repeats. In *T. gondii* six further proteins homolog to TgOWP1 were identified and characterised [6]. They all lack type II domains and two of them (TgOWP2 and TgOWP4) have so called degenerated type I repeats. Three of these proteins (TgOWP1, TgOWP2, and TgOWP3) had been characterized further, but the results [6] are not free from contradictions. These TgOWP 1–3 genes had been amplified from cDNA derived from partially sporulated oocyst, but the polyclonal antibodies produced against these proteins, localised to the oocyst wall [6], which is already formed before sporulation process begins [20]. However, TgOWP2 and TgOWP6 are described to be present in the proteome of the sporocyst fraction [7] and support the discrepancies concerning the localisation of TgOWP2 in *T. gondii.* The existence of COWP, respectively TgOWP, homolog proteins in *Eimeria* was assumed by several authors [21, 22, 8] and recently confirmed [23]. To study COWP and TgOWP homolog proteins in *Eimeria nieschulzi* we presumed to utilise reporter gene assay as a suitable tool. This technique facilitates to detect time point and place of protein expression at once, and prevents the risk of interference of cross reactions like in antibody based assays, which can be expected if several structural similar proteins are expressed in the same organism. The stable transformation of *Eimeria* parasites is a relatively new approach [9–12] which allows expression of reporter protein in the parasite throughout the life cycle. Until now, there was only one study which had used this technology to track autologous proteins during the sporulation process [24]. We used the pyrimethamine resistant, fluorescent DHFR/TS-YFP molecule [25, 12], for selection and visual control of successful transformation and expected a coupled positive selection of adjacent reporter cassettes, as seen in previous experiments [12]. Under control of the putative EnOWP2 promoter (construct1), we could see the expression of mCherry during the sporulation in the parasites cytoplasm. This indicated the upregulation of EnOWP2 during sporulation and was also confirmed by the transcript analysis. For the corresponding gene in *E. tenella,* EnOWP2*,* this was also affirmed [23]. The cytoplasmatic expression was expectable through the absence of any theoretical signal sequences prompting a specific transport. In fully sporulated oocyst mCherry was predominantly found in multiple point shaped bodies or vesicles in the sporocyst residuum. *Eimeria nieschulzi*, transfected with construct 2 (mCherry fused to the EnOWP2-gene) showed different reporter signals, but in the same time frame like the EnOWP2-promoter driven mCherry. The mCherry reporter could only be functionally translated if the transformed parasites splice the primary transcript correctly. The mCherry reporter signal appeared initially, in the unstructured, empty appearing, extracytoplasmatic space between cytoplasm and oocysts wall and later predominantly co-localised with the sporocyst wall in fully sporulated oocysts.

These observations are comparable to ultrastructural observations made during the sporulation of *Eimeria brunetti* [20]. In this avian parasite, they found, that the sporocyst wall was formed during the formation of the sporozoites and the sporocyst wall formation was not associated with cytoplasmatic organelles like wall forming bodies, as it is known for the oocyst wall formation [1]. Further, it was observed that sporocyst wall and Stieda body are formed by “gradual coalescence of material onto the outer limiting membrane of the developing sporocyst” [20]. These ultrastructural findings are in accordance with our observation of the COWP2-mCherry fusion protein (similar also to COWP6-mCherry). Based on our data we suggest that COWP2 and COWP6 are expressed during sporulation and are exported and deposited in the extracytoplasmatic space between cytoplasm and oocyst wall. Due to the lack of a functional importance, this compartment was unnamed before and we designated it as ‘circumplasm’, because it is not just an empty space within the oocyst, but it is, at least temporarily, filled with proteins that play a role in sporocyst wall formation among other potential functions of this compartment that have yet to be researched. During sporozoite formation the EnOWP2-mCherry accumulates predominantly at one end, probably marking the early Stieda body, and within few hours it accumulates around the sporocyst, co-localising with the sporocyst wall. Confocal fluorescence microscopy shows that mCherry signals indeed limit the sporocyst, but it can also be found embedding the sporozoites and few signals were also found in the sporocyst residuum. The excystation experiments carried out in this study, confirm light and electron microscopic observations made with rodent *Eimeria* species [26]. Under the influence of excystation medium the Stieda body degraded very fast and left a hole for sporozoite’s exit. The red fluorescence signals, initially still visible at sporocyst wall, disappeared after exit of the sporozoites. We interpret this observation, that the Stieda body region is the trypsin/bile susceptible structure of the sporocyst, whereas the sporocyst wall is firstly protected from external proteolytic degradation. After exit of the sporozoites, the proteolytic trypsin can simultaneously enter the sporocyst and degrades the sporocyst wall, indicated by the disappearing mCherry signal. For some avian *Eimeria* species, the degradation of sporocyst walls under influence of trypsin had also been described [27] and supports our observations and interpretation. Analogous observations were made for the oocyst wall of *E. nieschulzi*. In this case, pepsin could only enter the oocyst at the polar caps for the degradation of the inner oocyst wall [28]. Thus, another protecting structure is limiting the EnOWP2 and EnOWP6 containing sporocyst wall, as the immunofluorescence antibody detection of mCherry in the sporocyst wall suggest. Therewith, probably the *E. nieschulzi* sporocyst wall has an outer membrane boundary. A limiting membrane onto the sporocyst wall of *Eimeria larimerensis,* also a rodent *Eimeria* species, had been described [26], but no ultrastructural study about *E. nieschulzi* had focused on that. For the process of sporocyst wall formation the existence of a limiting membrane has consequences. Either the EnOWP2 and EnOWP6 are cross linked on the outer limiting membrane, like previously observed [20] and an additional membrane is built later (respectively during this process), or a membrane is already existing and both proteins are imported back from the circumplasm. This data supports that membranes seem to play a role in sporocyst formation. Their precise function needs to be elucidated. An appreciable expression of EnOWP 2 and EnOWP6 in the sporocyst can be probably excluded, because in transgenic oocyst all sporocyst walls show the red fluorescence, even if not all sporocysts (respectively the sporozoites inside) showed YFP expression. The selection of transformed parasites in the host animal is not absolute and by this reason cross fertilization of wild type with transformed parasites can occur [11]. We suspect that during the back crossing the plasmid supplied EnOWP2 and EnOWP6 alleles are present in the zygote and corresponding proteins became expressed and transported to the circumplasm before meiosis. After meiotic division the alleles became distributed to the haploid sporocyst. The mCherry signal was found always in all sporocyst walls of the oocyst, but the YFP signals followed predominantly Mendelian inheritance. Phenotypes with YFP in three or one sporocysts per oocyst are conceivable of multiple integrations occurred, or inserted DNA is not stable during meiosis as discussed before [11]. For the export of EnOWP2 and EnOWP6 we hypothesise a transport via the secretory pathway. In both proteins, signal peptides were predicted by the SignalP4.0 prediction tool at the N-terminal end. During the transport, the fused mCherry fluorochrome is probably not functional and became first correctly folded and visible in the extracytoplasmatic space (cicrumplasm). This hypothesis is supported by EnOWP2exon1-mC reporter (construct 3). In this case mCherry also exported to the circumplasmatic space and had a predominantly vesicle like distribution in the cytoplasma. Due to the lack of the majority of cysteine residues, the reporter is probably correctly folded and appeared visible during the transport. The amino acid sequence encoded by EnOWP2exon1 seems to be suitable for the export and also for the import during sporocyst formation, because mCherry can be seen in fully sporulated oocysts, between sporocyst wall and the sporozoites, but it did not co-localise with the sporocyst wall. Additionally the mCherry was found in the sporocyst residuum, and was absent from the circumplasm. This supports an assumption, that cysteine residues are responsible for the integration in the sporocyst wall. It was previously assumed that cysteine residues of this protein class (COWP) form intermolecular disulfide bonds which were hypothesized for COWPs in *Cryptosporidium* [5]. The mCherry, fused to EnOWP2 or EnOWP6 genes under control of the particular promoter, was found in both cases co-localised with the sporocyst wall of fully sporulated oocyst, but there are also some differences in time of expression and signal patterns. The EnOWP6-mCherry reporter was observed predominantly six hours after isolation from the host in the circumplasm. In some oocysts we observed expression in the emerging gap, between cytoplasm and inner oocyst wall. An expression in macrogamonts or at the outer oocyst wall was not observed. In contrast, EnOWP2-mCherry could not be observed earlier than 12 h post isolation and the signals were co-localised with the sporocyst wall in sporulated oocysts as well as in a concentrate at the polar ends of the sporocyst, probably in a structure known as Stieda body, respectively also in a putative cryptic Parastieda body at the opposite end of the sporocyst. The existence of a Parastieda body has been described for other rodent *Eimeria* species [29], but we found no morphological evidence for Parastieda body in *E. nieschulzi* by light or scanning electron microscopy (not shown). The EnOWP6-mCherry reporter signals are regularly distributed to sporocyst wall and some of the reporter signals remained in the circumplasm of fully sporulated oocysts, whereas mCherry of EnOWP2 reporter was almost absent at this stage.

### Conservation of EnOWP2 and EnOWP6 within Coccidia

In our study we found EnOWP2 as an orthologous protein to TgOWP2 from *Toxoplasma gondii*. This is supported by similar intron-exon structure of both genes, the nearly identical number of encoded amino-acid, similar time of expression, and as well as the same degenerated Type I repeats with similar distances between the cysteine residues.

In *T. gondii* TgOWP2 has been described as a protein of the oocyst wall [6] but not of the sporocyst wall. In conflict to this observation, transcripts of TgOWP2 can be found in the unsporulated oocysts [6] and the proteome of the sporocyst fraction includes TgOWP2 and also TgOWP6 [7]. For the immunodetection of TgOWP2 [6] no membrane permeabilising reagent was used, whereas in the successful detection of the GAM22 protein in the *E. necatrix* sporocyst wall Triton X-100 was applied [30]. Further, Anti-TgOWP2-Antibodies had a cross reactivity to TgOWP1 and TgOWP3 [6], two proteins which are probably indeed proteins of the oocyst wall in *T. gondii*. It is conceivable that TgOWP2 also plays a role in the sporocyst wall formation in *T. gondii* and should be reinvestigated.

In different *Eimeria* species we found EnOWP2 highly conserved in the genome data. The gene predictions of EnOWP2 orthologs in *Eimeria acervulina* and E. maxima were in accordance with our data. For the other avian *Eimeria* species and mouse parasite *E. falciformis* we changed the predictions according to our data, because we could find the same intron-exon structure for this gene in all investigated *Eimeria* species. The *Eimeria* COWP2 homolog in *E. tenella*, *E.acervulina and E. maxima* is expressed during sporulation, and was not found in the gametocyte stage of *E. tenella* in a recent study [23] which confirms our data. Hence we expect the same, conserved function for this protein in *Eimeria*.

However, a conserved function of the other investigated gene product EnOWP6 is much more difficult to evaluate. The homologous in *T. gondii* TgOWP6 was found in the proteome of the sporocyst fraction [7] and transcripts were found in sporulating oocyst, but no further data concerning its localisation are available in *T. gondii*. Recently, an ortholog from *E. tenella,* named EtOWP6, was found highly upregulated in gametocytes, and expressed in unsporulated oocysts as well [23].

Polyclonal antibody serum, directed against a peptide, labelled the wall forming bodies I in the macrogamonts but no other structures or stages [23]. Sequence comparison reveals that *E. tenella* and *E. necatrix* have different N-terminal protein sequences, compared to those from *E. nieschulzi* and *E. falciformis*, parasitizing in rodents. This fact may reflect another or dual role in avian *Eimeria* species compared to *E. nieschulzi* because we could not amplify EnOWP6 from gametocyte cDNA and the reporter protein was only visible during the sporulation, but not in the gametocyte stage.

Based on our data and the expression profiles from other *Eimeria* species we assume that at least one function of the *Eimeria* COWP6 ortholog, is the participation in the sporocyst wall formation as a structural protein. But also further, different functions in different *Eimeria* species are conceivable because all oocysts are apparently similar, but the oocyst of *E. nieschulzi* are protease sensitive and harbour cap structure [28] which is not the case in avian *Eimeria* species.

### Ethics, consent and permissions

The authors declare that the experiments comply with the current laws of Germany where they were performed.

Experiments with animals were registered at Regierungspräsidium Dresden (Reference Numbers 24–9168.25–8–2004–1 and 24D–9168.25–8–2006–1). Production of genetically modified parasites was registered at Saxon State Ministry of the Environment and Agriculture (Reference Number 55–8811.72/58). The handling of genetically modified parasites at the BNI in Hamburg was registered at the Behörde für Stadtentwicklung und Umwelt, Amt für Immissionsschutz und Betriebe (Reference Number E34–213/03).

## Conclusions

In summary, we think the experiments performed in this study disclose plausible evidence for the function of the COWP homologous proteins EnOWP2 and EnOWP6 from *E. nieschulzi* as sporocyst wall proteins. A similar function of the correspondent homologous OWPs can be assumed also in other *Eimeria* species and *Toxoplasma gondii*. Beyond that, the dynamics of the reporter proteins during the sporulation allow us to get an idea how the sporocyst wall is formed without wall forming bodies, but under participation of a so far uncharacterized compartment.

## Additional files

Additional file 1:
**Primer sequences and annealing temperatures.**
Additional file 2:
**Paragraph A)** ***Eimeria*** **COWP2 genomic and cDNA sequences.** Paragraph B) Intron-Exon structure of *Eimeria* COWP2 orthologs. Paragraph C) BLAST results of EnOWP2 and EnOWP6 vs. TgOWPs. Paragraph D) Alignment of TgOWP2 and EnOWP2 .Paragraph E) Analysis of orthologs of EnOWP2 and EnOWP6 in ESTs of related Apicomplexa. Paragraph F) Sequence of EnOWP6. Paragraph G) Alignment of TgOWP6 and EnOWP6. Paragraph H) Alignment of EnOWP6 with ToxoDB entries of other *Eimeria* species. Additional file 3: Figure S2. Stage specific expression EnOWP2 and EnOWP6. Transcripts of EnOWP2 (Lane 5) and EnOWP6 (Lane 14) had been amplified from cDNA of sporulating oocyst (24 h), but not from sporozoite cDNA (Lane 3 and 12) or cDNA derived from gamonts (Lane 4 and 13). From genomic DNA both intron harbouring genes had been amplified (Lane 2 and Lane 11), which resulted in longer amplification products than from cDNA. To ensure gamont cDNA quality the gametocyte specific gene EnGam82 had been utilized and was successfully amplified von gamont cDNA (Lane 9). Further small ribosomal subunit gene was successfully amplified from genomic DNA and sporozoite cDNA which reveals sufficient template quality. Additional file 4: Figure S1. Distribution of YFP and mCherry signal in pEnOWP2Pro-mC and pEnOWP2Pro-mC. The different distribution of fluorescence signal within these transgenic lines is visualized in this figure. The YFP signal alters in sporulated oocysts and one, two, three, or four sporocysts, harbouring YFP expressing sporozoites, were observed in the single oocysts. The mCherry signals were distributed to all sporocyst and did not alter. See also Additional file 5 and Additional file 6. For quantifications see Table 1. Additional file 5:
**3D-Animation of mCherry and YFP signal in pEnOWP2-mC transformed oocyst, corresponding to Fig.** 7a **.**
Additional file 6:
**3D-Animation of mCherry (red) and YFP (green) signals in pEnOWP2-mC transformed oocyst, corresponding to Fig.** 7b **.**
Additional file 7: Figure S3. IFA with anti-mCherry antibody on wild type sporocysts. Excysted wild type sporocyst were MeOH fixed and permeabilised triton x-100 (A) or used unfixed and not permeabilised (B) as negative control for anti-mCherry antibody test. Neither the mCherry nor the FITC channel fluorescent signals were detectable independently of the fixation or permeabilisation. Scale bar 5 μm. TL: Transmitted light; mCherry: mCherry fluorescence channel, FITC: FITC fluorescence channel. Additional file 8: Figure S4. Gamonts of pEnOWP6mc transformed parasites (P1). At 172 h p.i. we could observe non fluorescent, and only YFP expressing macrogamonts, without mCherry signal indicating absence of EnOWP6 in the gamont stage. Scale bar 20 μm. A: Transmitted light B: mCherry channel C: YFP channel. D: Overlay of A,B and C. Additional file 9:
**Animation of virtual sections through a pEnOWP2-mC transformed oocyst.** red: mCherry, green: YFP, blue: oocyst wall.
