# Supplementary material for: Two COWP-like cysteine rich proteins from Eimeria nieschulzi (coccidia, apicomplexa) are expressed during sporulation and involved in the sporocyst wall formation
Source: Parasit Vectors. 2015 Jul 25;8:395. doi: 10.1186/s13071-015-0982-3 (PMC4514997; doi:10.1186/s13071-015-0982-3)
Supplement: Additional file 1: — Primer sequences and annealing temperatures. [file 13071_2015_982_MOESM1_ESM.doc]

Supplementary File 2

| No. | Sequence | Anneling Temp. |
| --- | --- | --- |
| #1 | tagatcaaagcttgcggccgcacaggaaatgcctctttctcc | 57°C |
| #2 | tagatccttaagcctaaagaaagaatttaaaac | 57°C |
| #3 | gatcaagcttgcggccgcatacgcaacgccggaatcatgg | 60°C |
| #4 | tagatccttaaggaaaatggggcgatagacac | 60°C |
| #5 | tagatccttaagtccgcagagggggggcaccatg | 60°C |
| #6 | tagatccttaagctgtgggtcaaactcagccgat | 60°C |
| #7 | atgggacacttggctgtttc | 60°C |
| #8 | ttagatgggttctcccctg | 60°C |
| #9 | atgaagtattgcatggttgc | 57°C |
| #10 | tcatccgcagagggggggca | 57°C |
| #11 | ctgcccactctggaaaatgc | 57°C |
| #12 | tgtaggtcgtttcccagg | 57°C |

Primer sequences and annealing temperatures
