# Supplementary material for: Two COWP-like cysteine rich proteins from Eimeria nieschulzi (coccidia, apicomplexa) are expressed during sporulation and involved in the sporocyst wall formation
Source: Parasit Vectors. 2015 Jul 25;8:395. doi: 10.1186/s13071-015-0982-3 (PMC4514997; doi:10.1186/s13071-015-0982-3)
Supplement: Additional file 2: — Paragraph A) Eimeria COWP2 genomic and cDNA sequences. Paragraph B) Intron-Exon structure of Eimeria COWP2 orthologs. Paragraph C) BLAST results of EnOWP2 and EnOWP6 vs. TgOWPs. Paragraph D) Alignment of TgOWP2 and EnOWP2 .Paragraph E) Analysis of orthologs of EnOWP2 and EnOWP6 in ESTs of related Apicomplexa. Paragraph F) Sequence of EnOWP6. Paragraph G) Alignment of TgOWP6 and EnOWP6. Paragraph H) Alignment of EnOWP6 with ToxoDB entries of other Eimeria species. [file 13071_2015_982_MOESM2_ESM.doc]

**A) EnOWP2 orthologous proteins in *Eimeria* species.**

*1. Eimeria acervulina* EAH_00033530 (toxodb.org) is in accord with our prediction

*2. Eimeria maxima* EMWEY_00029600 (toxodb.org) is in accord with our prediction

*3. Eimeria praecox* original genomic OWP2 locus of Eph_scaff17

with assembly gaps (Ns) GenBank: HG690782.1

**ATGAGACATT A**TATTAT**TGC TGTCACATTG TTTTGTTCGG CAGCGGGATC**

**TCGTTCCCTA GCCGTTGAAG ACGGCAATGC TACCGCAAAT GGCTTTGCAG**

**AAGCGACGAT GCGACGACTG AAAGAGATGC CCCCAACAAA GTGCGCATGT**

**CAAGAAGGAT ACACTCTTGC CGACGGAACG TGCATCCGGA CGACTGAAAG**

**TGAACCACAG GTAAACGGAA TTCACCCATA TGAATCTTTG TGAGCTCGCA**

**ACGTGGCTTT ATCCAGGAAA** TAT**GCCACAT *CGGGAACATG* GTGGACGGCC**

**TGTGTGTCAT GCCCGCACAA GCTGTGTTGC AATGTCCTGA TGAGTACATN**

**NNNNNNNNNC CAAGAAGGAT ACACTCTTGC CGACGGAACG TGCATCCGGA**

**CGACTGAAAG TGAACCACAG GTAAACGGAA TTCAACCATA TGAATCTTTG**

**TGAGCTCGCA ACGTGGCTTT ATCCAGGAAA** TAT**GTCACAT CGGGAACATG**

***GTGGACGGCC* TGTGTGTCAT GCCCGCACAA GCTGTGTTGC AATGTCCTGA**

**TGAGTACATC ACGGTTTGCA AGAAGAAAGA CACCGCAGAA TCACCCTGTT**

**GCGCCAAACC CCAGACGGCC GAGAGGATTG CTCGCTGCCC AGACGGAACT**

**GCGTTCTTGG AAGGCCACTG CACAAGGATT CTTGCCCACC GCCTTGTCGC**

**CGAATGCCCA CTTGGCTTTG GCCTTTCCGA ACATGGGACC CAATGCATCA**

**GAGAAGAACA AGGACCTCCT GCTCCAACTT GTGTGCCCCC GGACTTTCTG**

**TCACCGGAAG GAGATTCCTG CATAACAACA ACCGAGCAAG GATTTGAATA**

**CGTTTGCCCG GACGAG**TAT**G AGTGCATTTC TCACACCATC AAGAAGAAGA**

**AGAAATACAG TCCCCTGTGC TCGGCATGTG CCAAGACAAC GGAAGCGCCT**

**CCAACCTGTG GGTGTCCTGA AGGCCTGCAG GAGGTTGGCG GTTTCTGCTA**

**TGACCCAGAT ATTTATGCTC TCTGCCAAAC TCGAGCGCCA GCGCCGCGCA**

**AGCAAGCACC TTCCAANNNN NNNNNNACCT CCAACCTGTG GGTGTCCTGA**

**AGGCCTGCAG GAGGTTGGCG GCTTCTGCTA TGACCCAGAT ATTTATGCTC**

**TCTGCCAAAC TCGAGCGCCA GCGCCGCGCA AGCAAGCACC TTCCAAGTAC**

**CAAGCGTCAT ATCCATCAAA GGAAGCTCCA GAGCCAGAGA TCGACTGCTC**

**GCCGATCGGA TCCGTCACAT GCGATTGCAC ACTACCGTTC TCTCTCGAGT**

**GCAATGGAGA CGCGTGCAGA TGCCTTCACC GGCAGGTGCT TCCCACCATG**

**CCCATTTGCC GAGGGGAGAT TGACGAAGCC GGAAACTGTT TAACTCAAGC**

**CAAAAAGCGC CTCTTG**TAT**A CTTGCCCAGA AGGTTTTACA TGTGACGTCG**

**TGGACAAGAA GGGCCGCTGC GAATGTACGA GAATCGTAGT CGCTGAGCCT**

**ATCCCCAGAT GTCTCGCAGG AGAGCCCCAA GGCAGCAAGT GCATTGAAGC**

**CATCCAAGAA GAAAAGATTT TGGACTGTCC ACCTGGATAC ACGGAAAACT**

**GCTGTGAAGA TCAGTGCACA TGCACAAAGA CACACTTGGC CGTGAGGCAG**

**GTCAAGTGTG AAGAAGGTGC AGTAAGCATT CAAGGACAAT GCGCT**TAT**GT**

**AACGCAGCCC TCGCCCGGAT GCTACGAGGT AAGTTCTTGA ACACTG**TAT**T**

**GGCCGT**TAT**T TGCTCCAAAA GAAAACTCTC CCAGGGCGTG CTTCGAGGGG**

**ACAGATG**TAT **CCAGGAGTAC ATTGTACCGC CCACCTGCGG ATAA**

Assembly gaps (Ns) are within duplicated sequences,probably caused by mis-assembly.

Duplicated sequences are with red and pink backround color. For correction of the genomic EpOWP2-locus the duplicated sequences (white coloured characters) were removed (see corrected sequence below).

*Eimeria praecox* corrected genomic OWP2 locus with introns and exons

**ATGAGACATT ATATTATTGC TGTCACATTG TTTTGTTCGG CAGCGGGATC**

**TCGTTCCCTA GCCGTTGAAG ACGGCAATGC TACCGCAAAT GGCTTTGCAG**

**AAGCGACGAT GCGACGACTG AAAGAGATGC CCCCAACAAA GTGCGCATGT**

**CAAGAAGGAT ACACTCTTGC CGACGGAACG TGCATCCGGA CGACTGAAAG**

**TGAACCACAG GTAAACGGAA TTCACCCATA TGAATCTTTG TGAGCTCGCA**

**ACGTGGCTTT ATCCAGGAAA** TAT**GCCACAT CGGGAACATG GTGGACGGCC**

**TGTGTGTCAT GCCCGCACAA GCTGTGTTGC AATGTCCTGA TGAGTACATC**

**ACGGTTTGCA AGAAGAAAGA CACCGCAGAA TCACCCTGTT GCGCCAAACC**

**CCAGACGGCC GAGAGGATTG CTCGCTGCCC AGACGGAACT GCGTTCTTGG**

**AAGGCCACTG CACAAGGATT CTTGCCCACC GCCTTGTCGC CGAATGCCCA**

**CTTGGCTTTG GCCTTTCCGA ACATGGGACC CAATGCATCA GAGAAGAACA**

**AGGACCTCCT GCTCCAACTT GTGTGCCCCC GGACTTTCTG TCACCGGAAG**

**GAGATTCCTG CATAACAACA ACCGAGCAAG GATTTGAATA CGTTTGCCCG**

**GACGAG**TAT**G AGTGCATTTC TCACACCATC AAGAAGAAGA AGAAATACAG**

**TCCCCTGTGC TCGGCATGTG CCAAGACAAC GGAAGCGCCT CCAACCTGTG**

**GGTGTCCTGA AGGCCTGCAG GAGGTTGGCG GTTTCTGCTA TGACCCAGAT**

**ATT**TAT**GCTC TCTGCCAAAC TCGAGCGCCA GCGCCGCGCA AGCAAGCACC**

**TTCCAAGTAC CAAGCGTCAT ATCCATCAAA GGAAGCTCCA GAGCCAGAGA**

**TCGACTGCTC GCCGATCGGA TCCGTCACAT GCGATTGCAC ACTACCGTTC**

**TCTCTCGAGT GCAATGGAGA CGCGTGCAGA TGCCTTCACC GGCAGGTGCT**

**TCCCACCATG CCCATTTGCC GAGGGGAGAT TGACGAAGCC GGAAACTGTT**

**TAACTCAAGC CAAAAAGCGC CTCTTGTATA CTTGCCCAGA AGGTTTTACA**

**TGTGACGTCG TGGACAAGAA GGGCCGCTGC GAATGTACGA GAATCGTAGT**

**CGCTGAGCCT ATCCCCAGAT GTCTCGCAGG AGAGCCCCAA GGCAGCAAGT**

**GCATTGAAGC CATCCAAGAA GAAAAGATTT TGGACTGTCC ACCTGGATAC**

**ACGGAAAACT GCTGTGAAGA TCAGTGCACA TGCACAAAGA CACACTTGGC**

**CGTGAGGCAG GTCAAGTGTG AAGAAGGTGC AGTAAGCATT CAAGGACAAT**

**GCGCTTATGT AACGCAGCCC TCGCCCGGAT GCTACGAGGT AAGTTCTTGA**

**ACACTGTATT GGCCGTTATT TGCTCCAAAA GAAAACTCTC CCAGGGCGTG**

**CTTCGAGGGG ACAGATGTAT CCAGGAGTAC ATTGTACCGC CCACCTGCGG**

**ATAA**

exon region: green, intron region: grey

putative protein coding sequence in original Eph*_*scaff17

by joining of bp 29771...29980, 30247…30786, 31498…31555

**putative AS sequence of *E. praecox* OWP2 (EpOWP2)**

**MRHYIIAVTLFCSAAGSRSLAVEDGNATANGFAEATMRRLKEMPPTKCAC 50**

**QEGYTLADGTCIRTTESEPQEICHIGNMVDGLCVMPAQAVLQCPDEYITV 100**

**CKKKDTAESPCCAKPQTAERIARCPDGTAFLEGHCTRILAHRLVAECPLG 150**

**FGLSEHGTQCIREEQGPPAPTCVPPDFLSPEGDSCITTTEQGFEYVCPDE 200**

**YECISHTIKKKKKYSPLCSACAKTTEAPPTCGCPEGLQEVGGFCYDPDIY 250**

**ALCQTRAPAPRKQAPSKYQASYPSKEAPEPEIDCSPIGSVTCDCTLPFSL 300**

**ECNGDACRCLHRQVLPTMPICRGEIDEAGNCLTQAKKRLLYTCPEGFTCD 350**

**VVDKKGRCECTRIVVAEPIPRCLAGEPQGSKCIEAIQEEKILDCPPGYTE 400**

**NCCEDQCTCTKTHLAVRQVKCEEGAVSIQGQCAYVTQPSPGCYEGVLRGD 450**

**RCIQEYIVPPTCG**

*4. Eimeria brunetti* OWP2 locus with introns and exons in scaffold

Ebh_scaff20 (GenBank: HG711107.1)

**ATGAGGCATT ATATTATTGC CGTCACATTC TTTTGTTCGG CCACGGGGTC**

**TCGCTCCCAA ACGGCTGCAG ACGGAGATGC TAGCGCAAAA GTCTTTGCAG**

**GGGCGGCAAT GCGGCAACTG AAAGCGATGC CCCCCGCAAA GTGCATGTGC**

**CAAGATGGAT ACACTCTCAT AGACGGAACC TGCATCATGA CGATTGAAAG**

**TCTACCCCAG GTAAACGGAC TTTGATCGCA CCATATTTTT ATTCGCTTGT**

**AACGAGGCTC CTTTCCAGGA AACATGCCCA ATAGGGAACA TGGTGGACGG**

**CCTCTGTCTC TCGCCCGCAC AAGCCGTGCT GCAGTGTCCT GAACACTACA**

**TCACGGTTTG CGATAAGAAA GACTTGGTAG AATCACCCTG CTGCGCCAAA**

**GCACAGACGG CCGAGAAGAT TGCTCGCTGT CCAAACGGAA CGGAGTTCCT**

**GGATGGCTCT TGCACAAGGT TTCTTACCCA CCGACCCATC GCAGAATGCC**

**CTCTTGGTTT TGGTCTTAAC GAACATGGAA CTCAGTGCTT CAGAGAAGAA**

**ATCGGAGCGC CCGCTCCACT TTGTGTTCCA CCGGATGTTA TGTCACCGGA**

**GGGAGATGGA TGCATAACAC CAACAGAGCA AGGATTTGAA TACGTTTGCC**

**CGGACGAGTA TCACTGCATT TCTCACAACT TAAAGAAGAA GAAACACAGC**

**AAATACAGTC CACTGTGTTC CGCATGCGCC AAGACAGCGG AGGCACCGCC**

**ACGCTGTGGA TGCCCTCAAG GACTACAGGA GGTTGGCGGC TTCTGCTACG**

**ACCCAGATAT TTATGCTCTG TGCCAAAATC GAAGAGCAGT CCCGCGAAAG**

**CAAGCACCCT CCAAGAAAGC GGCACCGGCG CCATATCCAT CAAAAGACGC**

**TCCGGAGCCA GTGATCGACT GCAAGCCAGT TGGTCCTGTC GCATGCGAAT**

**GTCAACTTCC GTTCTCCCTC GAATGCAACG GAGACCTTTG CAGATGTCTT**

**CACCGGCTTG TGCTCCCCAC GACTCCCATC TGCAGAGGAC AGATTGACGA**

**TGCAAACAAC TGCATTGCTC TAGCCAAAAA GCCCCTTGCG TATACTTGCC**

**CAGAAGGCTT CACATGCGAT GTCGTGGGCA AGAAGGGCGA GTGCCGTTGT**

**ACCCGAATCA TAGTCGCCGA GCCCATCTCG AGATGTCGCA TTGGACAGCC**

**CCACGAAGGC GGTTGCATTG AAGTCATCAA AGAAGAAAAA GTTCTTGACT**

**GCCCGCCTGG ATACTTTGAA AACTGCTGTG ACGGCATATG CACCTGTACG**

**AAGACGCACT TGGCCGTACG ACAGGTCAAG TGTGAAGAAG GTGCTGTCAG**

**CATTCAAGGA CACTGCGCTT ACGTAAACAA ACCCGCACCC GGGTGCTTCG**

**AGGTAAGTTA CTATGCATTG TATATGCTCC AAGCCGTCCC TAACGCAAAC**

**AATCGCAGGG TGTGCTGCGA GGGGAGAGAT GCGTCCAGGA GGTCATCGTA**

**CCGCCCAGCT GTGGGTGAAG TTTGA**

exon region green, intron region grey

putative protein coding sequence in Ebh_scaff20

by joining of bp 125068…125277,125336…126469,126526…126585

**putative AS sequence of *E. brunetti* OWP2 (EbOWP2)**

**MRHYIIAVTFFCSATGSRSQTAADGDASAKVFAGAAMRQLKAMPPAKCMC 50**

**QDGYTLIDGTCIMTIESLPQETCPIGNMVDGLCLSPAQAVLQCPEHYITV 100**

**CDKKDLVESPCCAKAQTAEKIARCPNGTEFLDGSCTRFLTHRPIAECPLG 150**

**FGLNEHGTQCFREEIGAPAPLCVPPDVMSPEGDGCITPTEQGFEYVCPDE 200**

**YHCISHNLKKKKHSKYSPLCSACAKTAEAPPRCGCPQGLQEVGGFCYDPD 250**

**IYALCQNRRAVPRKQAPSKKAAPAPYPSKDAPEPVIDCKPVGPVACECQL 300**

**PFSLECNGDLCRCLHRLVLPTTPICRGQIDDANNCIALAKKPLAYTCPEG 350**

**FTCDVVGKKGECRCTRIIVAEPISRCRIGQPHEGGCIEVIKEEKVLDCPP 400**

**GYFENCCDGICTCTKTHLAVRQVKCEEGAVSIQGHCAYVNKPAPGCFEGV 450**

**LRGERCVQEVIVPPSCG***

*5. Eimeria mitis* OWP2 locus with introns and exons in scaffold

Emh_scaff164 (GenBank: HG681376.1)

**ATGAGGCCTT ATATTATTGC TGTCACACTG TTTTGTTCGG CCACGGGGTC**

**TCGCTCCCTA ACAGCTGCAG ACGGCGATGC TAACGCAGAT GGCCTTGCAG**

**AGTCGGTGAC GCGACAACTC AAAGCGATGC CACCAGCAAA GTGCATCTGC**

**AAAGATGGAT ACACTCTCAC TGACGGAACG TGCATCCGGA CAATGGAAAG**

**TGAACCTCAG GTAAATAAAA GTTGACTATG TCAAGCTGTG TACGTTCGTA**

**ATGCGGTTCC ATTTCAGGAA ATATGCCCAG TCGGAAACGT GGTAGACGGT**

**CTCTGTATGC TGCCAGCACA AGCTGTGCTG CAGTGCCCTG ACCACTACAC**

**CACGGTTTGC AAGAAGAAAG ACAGAGTAGA ATCACCTTGC TGCGCCAAAG**

**CAGAGATGGC AGAGAAGATT GCTCGCTGTC CAGACGAGAC TGAGTTCAAG**

**GATGGCCACT GCATAAGGGT TATTACCCAC AAACCCGTCT CCGAATGTCC**

**ACTTGGTTTT GGCCTTTCTG AGCACGGAAC TCAGTGCATC AAAGAAGAAC**

**TTGGAATGCC TGCTCCTCTT TGCGTTCCTC CGGACGTTAT GTCACCGGAA**

**GGAGATTCCT GCATAACCGT GACCGAGCAA GGGTTTGAAT ACGTCTGTCC**

**GGACGATTAT CAGTGCATTT CCCACACCAT CAAGAAGGCG AAGAAATACA**

**GCCCGCTGTG TTCCGCATGC GCCAAGACAA CGGAGGCACC GCCACTCTGC**

**GGATGCCCTG AAGGCCTGCA GGAGGTTAAC GGCTCCTGCT ATGATCCAGC**

**GATTTATGCT CTCTGTCAAA CTCGAGTGGC AGCCCCACGA AAGCAAGCAC**

**CGTCCAAGAA ACAGGCGGCA TACCCATCAA AAGAAGCTCC AGAGCCAGTG**

**ATCGACTGCA AGCCGATCAG CGCAGTCGCG TGCGAATGTC AACCTCCATT**

**CTCGCTCGAA TGTGCCGGAG ATGTGTGCAG ATGCCTCCAC CGGCTAGTTC**

**TTCCAACGAT GCCCGTATGC AGAGGAGAGA TCGACCCAGC CGGGAACTGC**

**CTAACCCCAG CCAAAACGCG CCTTTTGTAT ACTTGCCCAG AAGGCTTCAC**

**GTGCGACGTC GTGGACAAAA AGGGCCACTG CCGCTGTACA CGGGTCATAG**

**TCGCGGATCC CATCCCCAGA TGTCTCGTAG GAGAGCCCCA CGACACCAGA**

**TGCATTGAGG TCATCAGAGA AGAAAAGATC TTGGACTGCC CACCCGGATT**

**CACCGAAAGT TGCTGTGACA ATCAGTGCAC CTGCACGAGG ACACACTTGG**

**CCGTAAGACA GGTCAAGTGT GAAGATGGTG CAGTGAGCAT TCAAGGGCAG**

**TGCGCTTACG TATCGAAACC TTCTCTCGGA TGCTACGAAG TAAGTTTCTA**

**CGCATTTTAT GTGCTGTTAA TCTTTCCAAA CAAACGCCGT TCCAGGGTAT**

**GCTTCGAGGG GATAGATGCG TCCAAGAGGT CATCGTACCG CCCACCTGCG**

**GATAG**

exon region green, intron region grey

putative protein coding sequence in Emh_scaff164

by joining of bp 9218…9427, 9485…10606,10663…10722

**putative AS sequence of *E. mitis* COWP2 (EmiOWP2)**

MRPYIIAVTLFCSATGSRSLTAADGDANADGLAESVTRQLKAMPPAKCIC 50

KDGYTLTDGTCIRTMESEPQEICPVGNVVDGLCMLPAQAVLQCPDHYTTV 100

CKKKDRVESPCCAKAEMAEKIARCPDETEFKDGHCIRVITHKPVSECPLG 150

FGLSEHGTQCIKEELGMPAPLCVPPDVMSPEGDSCITVTEQGFEYVCPDD 200

YQCISHTIKKAKKYSPLCSACAKTTEAPPLCGCPEGLQEVNGSCYDPAIY 250

ALCQTRVAAPRKQAPSKKQAAYPSKEAPEPVIDCKPISAVACECQPPFSL 300

ECAGDVCRCLHRLVLPTMPVCRGEIDPAGNCLTPAKTRLLYTCPEGFTCD 350

VVDKKGHCRCTRVIVADPIPRCLVGEPHDTRCIEVIREEKILDCPPGFTE 400

SCCDNQCTCTRTHLAVRQVKCEDGAVSIQGQCAYVSKPSLGCYEGMLRGD 450

RCVQEVIVPPTCG*

# *6. Eimeria necatrix* OWP2-locus (reverse) with introns and exons in scaffold **Enh_scaff88 (GenBank: HG723015.1)**

**ATGAGGAACT ACACAATCGC TATCGCCCTG TTTTGTTCGG CCACAGGGTC**

**ACGTTCCCTA CCGGCTGGAG ACAATGCTGC CGCAGATGGG TTCGCAGAGA**

**CGCTGCTACG GAAATTGAAA GTCATGCCGC CTGCCAAGTG CACCTGCCAA**

**GATGGATTCA CCCTCATCGA CGGCACGTGC ACTCGGTCAA TTGAAACTGA**

**CCCACAGGTA AATTGATTGA CGGAGCTTGA CAAACTCAAA TTTCTTGCAT**

**TAATGTGTTT TCTGTGCAGG AAGTATGCCA CATTGGAAGC ATGGTGGAAG**

**GCCTTTGCGT CATGCCTGCA GAGGCAGTGA TGCAATGCCC CGACGAATAC**

**ATCACGGCTT GCACCAAAAA GGATTTAGCC CAATCACCAT GCTGCGCCAA**

**AGCACAGACG GCTGAGAAGA TGGCGCATTG CAGAGATGGA ACTGAGTTTC**

**ACGACGGCCA CTGCACGAGG GTTCTTACCC ACCAACCCGT GGTTGAGTGC**

**CCACTTGGCT TTGGTCTGTC GCATGACGGG CTTCTGTGCG TCAAGGAGGA**

**AATTGGACAG CCTACTCCAG TGTGTGCGCC GCCGGACGCC CTCTCAGCAG**

**AAGGGGATTC GTGTATAACA CCAATAGAGC AGGGCTTCGA ATATGTCTGC**

**CCTGACGAAT TCGAATGTAT TGCAAGTACT CACAAGAAAA AGAAATACAG**

**CCCGCTCTGT TCTGCGTGTG CTAGGACAAC GGAAGCACCG CCACACTGTG**

**GATGTCCTGA AGGCCAGATT GCGGTCGAGG GTTTCTGCTA TGATGCGGAT**

**ACGTACGCTC TGTGTCAAAA TCGCAGGGTT CCCCCGCGCA AGCAAGCGCC**

**ATCCAAAAAG CAGCCCGTGG CATACCCGTC AAAGGATATT CCAGAGCCAG**

**AAATCGACTG TAAACCAATC GGGCCGATCA TATGCGACTG TGATCGTCCC**

**TTTTCACTTG AATGCGCCGG CGAGGTGTGC AGATGCCTCC ACAGAGAAGT**

**CCTCCCCGTC ACGCCCATTT GTAGAGGACA ACTTGATGAG GGCGGGAACT**

**GCATCGCCCT GGCTCAGAAG AGGCCCATGT ATACTTGTGC AGAAGGATTC**

**ACATGCGATG TCATTGATAA GAAGGGCCAG TGCCGATGCA CCCGAATGCT**

**AACCGCCGAG CCCACTTCCA GATGCGTAGT AGGAGAACCC CACGGACACA**

**AATGTATAGA GGTTGTTAAG GAGGAGAAGA TTTTCGATTG CCCACCTGGA**

**TACATCGAGA CGTGCTGTGA AGAAGGGTGC ACCTGCACGA AGACACACTT**

**GGCCATGCGA CAGGTCAAAT GTGAAGAAGG TGCAGTGAGC ATCCAAGGCG**

**ATTGCGCTTT CGTTTCCAAG CCATCCGCAG GATGCTACGA GGTAACTAGT**

**TGAATTAATG CTTAAATAAA TCTCGGTACA AAATGAAACA ACCCCAGGGC**

**ATGCTACGAG GAGCAAAATG CGTCCAGGAC TTCATGGTGC CGCCACTCTG**

**CGGATGA**

exon region green, intron region grey

putative protein coding sequence in Enh_scaff88

by joining of bp 79458….79523; 79580…80701, 80764…80970

**putative AS sequence of *E. necatrix* OWP2 (EneOWP2)**

MRNYTIAIALFCSATGSRSLPAGDNAAADGFAETLLRKLKVMPPAKCTCQ 50

DGFTLIDGTCTRSIETDPQEVCHIGSMVEGLCVMPAEAVMQCPDEYITAC 100

TKKDLAQSPCCAKAQTAEKMAHCRDGTEFHDGHCTRVLTHQPVVECPLGF 150

GLSHDGLLCVKEEIGQPTPVCAPPDALSAEGDSCITPIEQGFEYVCPDEF 200

ECIASTHKKKKYSPLCSACARTTEAPPHCGCPEGQIAVEGFCYDADTYAL 250

CQNRRVPPRKQAPSKKQPVAYPSKDIPEPEIDCKPIGPIICDCDRPFSLE 300

CAGEVCRCLHREVLPVTPICRGQLDEGGNCIALAQKRPMYTCAEGFTCDV 350

IDKKGQCRCTRMLTAEPTSRCVVGEPHGHKCIEVVKEEKIFDCPPGYIET 400

CCEEGCTCTKTHLAMRQVKCEEGAVSIQGDCAFVSKPSAGCYEGMLRGAK 450

CVQDFMVPPLCG*

*7. Eimeria tenella* OWP2-locus (reverse) with introns and exons in scaffold Eth_scaff97 (GenBank: HG675767.1)

**ATGAGGAACT ACATTATCGC TATCGCCCTG TTTTGTTCGG CCACAGGGTC**

**ACGTTCCCTA CCGGCTGGAG ACAATGCTGC CGCAGACGGG TTCGCAGAGA**

**CGCTGCTACG GAAACTGAAA GTTATGCCGC CTGCCAAGTG CACCTGCCAA**

**GATGGATTCA CCCTCATCGA CGGCACGTGC ACTCGGTCAA TTGAAACTGA**

**CCCACAGGTA AATTGATTGA CGGAGCTTGA CAAACTCAAA TTTCGTGCAT**

**TAATGTGTTC CCTGTGCAGG AAGTATGCCA CATTGGAAGC ATGGTGGAAG**

**GCCTTTGCGT CATGCCTGCA GAGGCAGTGA TGCAATGCCC CGAAGAATAT**

**ATCACGGCTT GCACCAAAAA GGATTTAGCC CAATCACCGT GTTGCGCCAA**

**ATCACAGACG GCTGAGAAGA TTGCGCATTG CAGAGATGGA ACAGAGTTTC**

**ACGAAGGCCA CTGCACGAGG GTTCTTACCC ACCAACCCGT GGTTGAGTGC**

**CCAGTTGGCT TTGGTCTCTC GCATGAGGGG CTTCTGTGCG TCAAGGAGGA**

**AATTGGACAG CCTACTCCAG TGTGTGCGCC GCCGGATGCC CTCTCGGCAG**

**AAGGAGATTC GTGTATAACA ACAGTAGAGC AGGGCTTCGA ATATGTCTGT**

**CCTGACGAAT TCGAATGTAT TGCAAGTACT CACAAGAAAA AGAAATACAG**

**CCCGCTCTGT TCTGCGTGCG CTAGGACAAC GGAAGCACCG CCACACTGTG**

**GCTGTCCTGA AGGACAGATT GCGGTCGAGG GGTTCTGCTA TGATGCGGAA**

**ACGTACGCCC TGTGTCAAAA TCGCAGGGTT CCCCCGCGCA AGCAAGCGCC**

**ATCCAAAAAG CAGCCTGTGG CATACCCGTC AAAGGATATT CCAGAGCCAG**

**AAATCGACTG TAAACCAATC GGACCGATCA TATGCGACTG TGATCGTCCC**

**TTTTCACTTG AATGCGCCGG CGAGGTGTGC AGATGCCTCC ACAGAGAAGT**

**CCTCCCCGTC ACGCCCATCT GTAGAGGACA ACTTGATGAG GGCGGGAACT**

**GCATCGCCCT GGCTCAGAAG AGGCCCATGT ATACTTGTGC AGAAGGATTC**

**ACATGCGACG TCATTGATAA GAAGGGCCAG TGCCGATGCA CCCGAATGCT**

**AACCGCCGAG CCCACTTCCA GATGCCTAGT AGGAGAACCC CACGGACACA**

**AATGTATAGA GGTTGTTAAG GAGGAGAAGA TTTTCGATTG CCCACCTGGA**

**TACATCGAGA CGTGCTGTGA AGATCGGTGC ACCTGCACGA AGACACACTT**

**GGCCATGCGA CAGGTCAAAT GCGAAGAAGG TGCAGTGAGC ATCCAAGGCG**

**ATTGCGCTTT CGTTTCCAAG CCATCCGCAG GATGCTACGA GGTAACTAGT**

**TGAATTAATG CTCAAATAAA TCTCGATACA AAATGAAACA ACCGCAGGGA**

**ATGCTACGAG GAGTAAAATG CATCCAGGAC TTCATGGTGC CGCCACTCTG**

**CGGATGA**

exon region green, intron region grey

putative protein coding sequence in Eth_scaff97

by joining of bp 118896….119102; 119165…120286, 120343…120402

**putative AS sequence of *E. tenella* COWP2 (EtOWP2)**

MRNYIIAIALFCSATGSRSLPAGDNAAADGFAETLLRKLKVMPPAKCTCQ 50

DGFTLIDGTCTRSIETDPQEVCHIGSMVEGLCVMPAEAVMQCPEEYITAC 100

TKKDLAQSPCCAKSQTAEKIAHCRDGTEFHEGHCTRVLTHQPVVECPVGF 150

GLSHEGLLCVKEEIGQPTPVCAPPDALSAEGDSCITTVEQGFEYVCPDEF 200

ECIASTHKKKKYSPLCSACARTTEAPPHCGCPEGQIAVEGFCYDAETYAL 250

CQNRRVPPRKQAPSKKQPVAYPSKDIPEPEIDCKPIGPIICDCDRPFSLE 300

CAGEVCRCLHREVLPVTPICRGQLDEGGNCIALAQKRPMYTCAEGFTCDV 350

IDKKGQCRCTRMLTAEPTSRCLVGEPHGHKCIEVVKEEKIFDCPPGYIET 400

CCEDRCTCTKTHLAMRQVKCEEGAVSIQGDCAFVSKPSAGCYEGMLRGVK 450

CIQDFMVPPLCG*

1. *Eimeria falciformis* OWP2-locus with in intron and exon in contig 2387

*( E. falciformis* BayerHaberkorn1970_ ToxoDB-11.0_Genome - EfaB_Contig_2387)

**ATGAAGTATT GCATGGTTGC TATAACCTTG TTTTGTTCGG CCTCAGGCTC**

**TCGTTCCCTT GAGGCTGGAG ACGACTATGC TACCACAGAT GGATTTACCG**

**AAGAGCTGCT CCGGCAACTC AAAGTAGTGC CAGTACCCCC TGCAAAATGC**

**CAGTGCCCAG AAGGATACGA AATGCGGAAT GGCTCCTGTA TCAGATCGGT**

**TGAGTTTGAC CCACAGGTAA AAACATGCTT GACCAGCTTT ACACTTGTTT**

**GTACTTAATT GTTGCTCTGT AGGATGTTTG CCGAAGCGGA AAAATGGTGG**

**ACGGCCTCTG TGTCACTCTA GCACCGCAAG TCCTTCAATG CCCGCACGAC**

**TACGTTACGG TCTGCAAGAA GAAAGACCGA TCGGAATCTC CATGCTGCGC**

**CAAAGGAGAG ACAGCTGAGA AGGTTGCTCG TTGTAAAGAC GGAACGGACA**

**GTCATGATGG ACGCTGCACG CGAATCCTTA CCCGTGAACG AGTCCATGAG**

**TGTCCTCCCG GCTATGCCCT TATGAGCTCC CACGGGACTC AGTGTATCAA**

**ACAAGAATAT GGAGATGCTG CCCCAGCATG TGTTTATCCC GACGAATTGT**

**CCCCCGAAGG AGATTCATGT CTGACAACAA TACAGCAAGG CTTCGAATAT**

**GTATGCCCTG ACGAGTATGA GTGCGTTGCG CGCTCTCTGA AGAAGAAGAA**

**GAAGTACAGT CCGTTATGCT CTGCGTGCGT CAAGACTGAG GAAGCACAGC**

**CCACGTGCGG CTGTCCTGAA GGCCAGGATG AGTTCAACGG CTTCTGCTAT**

**GAGGCAGGAA TGCATGAGTT TTGTCAGTCC CACAAGGGGC TACCTCAAAA**

**ACAAGCACCC CCAAGTAAAA AGGGGTACAC TGCAGCGGAA TTGTACGAAC**

**AACCGCCGGC GGTCAACTGC AAGCCTTTAG GGCATGTAAC ATGCACATGC**

**GATCCTCCGT TTATCCTTCA AGGCTCCGGC GGATCAAGCA TTTGCATTCA**

**CAGGGATCTC ATCCCCGCTG TGCCCATTTG CAGAGGGCAG ACCGACGAAG**

**ATGGAAATTG CATCGCCCAA GTGCAGAAGC GCGTGCTCTA CGAATGCGCT**

**GAAGGATTTA CATGCGACGT TGTGAACAAG AAGGGACGAT GCAACTGCGT**

**CCGACTTAGT GCTGTCGAGC CCACTAGTCG CTGCGCTTCC GGGGAGGAAC**

**ACGAAGACAA GTGCATTGAG GTCATAGAGG AACCAAAAAT ACTGGAATGC**

**CCGCAAGGAT ATTCTGAAAC GTGCTGTGAT AACATATGCA CCTGCACAAA**

**GACAACCTTG GCTGTGAGAG AGGTCAAATG CGCTCCGGGG GCAGTTAGCA**

**TCCAAGGAGA GTGCGCGTAT GTTTCCAAGC CATCGACCAG TTGCGACGGG**

**GTAAGTACTT CATTGCGCTT TGCTTTATCG TGTCTGAAAA AGACGTCATC**

**TCAGGGACAG CTACGAGGAG AAAAATGTAC CCAGGATTTC ATGGCGCCCC**

**CCCTTTGCGG ATGA**

exon region green, intron region grey

putative protein coding sequence in EfaB_Contig_2387

by joining of bp 333400….333615; 333672…334799, 334854…334913

**putative AS sequence of *E. falciformis* OWP2 (EfOWP2)**

**MKYCMVAITLFCSASGSRSLEAGDDYATTDGFTEELLRQLKVVPVPPAKC 50**

**QCPEGYEMRNGSCIRSVEFDPQDVCRSGKMVDGLCVTLAPQVLQCPHDYV 100**

**TVCKKKDRSESPCCAKGETAEKVARCKDGTDSHDGRCTRILTRERVHECP 150**

**PGYALMSSHGTQCIKQEYGDAAPACVYPDELSPEGDSCLTTIQQGFEYVC 200**

**PDEYECVARSLKKKKKYSPLCSACVKTEEAQPTCGCPEGQDEFNGFCYEA 250**

**GMHEFCQSHKGLPQKQAPPSKKGYTAAELYEQPPAVNCKPLGHVTCTCDP 300**

**PFILQGSGGSSICIHRDLIPAVPICRGQTDEDGNCIAQVQKRVLYECAEG 350**

**FTCDVVNKKGRCNCVRLSAVEPTSRCASGEEHEDKCIEVIEEPKILECPQ 400**

**GYSETCCDNICTCTKTTLAVREVKCAPGAVSIQGECAYVSKPSTSCDGGQ 450**

**LRGEKCTQDFMAPPLCG***

1. *Eimeria nieschulzi* OWP2 locus (inverted) with introns and exons in contig 2812 (GenBank ASM82694v1)

**ATGAAGTATT GCATGGTTGC TATAACCTTG TTTTGTTCGG TCTCAGGCTC**

**TCGTTCCCTT GAGGCTGGAG ACGACTATGC TACCACAGAT GGATTTACCG**

**AAGAGCTGCT CCGGCAACTC AAAGTAGTGC CAGTACCCCC TGCAAAATGC**

**CAGTGCCAAG AAGGATACGA CATGCGGAAT GGCTCCTGCA TCAGATCGGC**

**TGAGTTTGAC CCACAGGTAA AAACATGGCT GACCAGCTTT ACACTTTATA**

**CTTAATTTTT GCTCTGTAGG ATGTCTGCCG GAGCGGAAAA ATGGTGGACG**

**GCCTCTGTGT CACTCCAGCA CCGCAAGTCC TTCAATGCCC GCACGACTAT**

**GTTACCGTTT GCAAGAACAA AGACCGATCA GAGTCTCCGT GCTGCGCCAA**

**AGAAGAGACA GCTGAAAAGG CTGCTCGTTG TAGAGACGGA ACGGACAGTC**

**ATGATGGACG TTGCACGCGG ATCCTTGCCC GTGAACGAGT CCATGAGTGT**

**CCTCCTGGCC ATGCCCTTAT GAGTTCCCAC GGGACTCAGT GTATCAAACA**

**AGAATATGGA GAAGCTGCCC CAGCATGCGT TTATCCGGAT GAATTGTCCC**

**CCGAAGGAGA TTCATGTCTG ACAACAATAC AGCAAGGCTT TGAATATATA**

**TGCCCTGACG AGTATGAGTG CGTTGCGCGC TCTCTGAAGA AGAAGAAGAA**

**GTACAGTCCG TTATGCTCTG CGTGCGTCAA GATCGAGAAG GCACAGCCCA**

**CGTGCGGCTG TCCTGAAGGC CAGGATGAGT TCAACGGCTT CTGCTATGAG**

**GCAGGAACGT ATGAGTTTTG TCAGTCCCGC AAGGGGCTAC CACAAAAACA**

**AGCACCCCCA AGAAAAAAGG GGTACACGGC AGTGGAATTG TACGAACAAC**

**AGCCAGAGGT CAACTGCAAG CCTTTAGGGC ATGTCACGTG CACATGCGAT**

**CTTCCGTTTA GCCTTCAAGG CTCCGGCGAA TCAAGCACTT GCATCCACAG**

**GGATCTTATC CCAGCTGTGC CGATTTGCAG AGGGCAGACA GACGAAAATG**

**GAAATTGCAT CGCCCAAGTG CAGAAGCGCG TGCTCTACGA ATGTGCTGAA**

**GGATTCACAT GCGACGTTGT TAACAAAAAG GGACGATGCA ACTGCGTCCG**

**ACTTACTGCC GTGGAGCCCA CTAAACGGTG CGCTTCCGGA GAGGAACGCG**

**AAGACAAGTG TATTGAGATC ATCGAGGAGC CAAAAATACT CGAATGCCCG**

**CAAGGATATT CTGAGACGTG CTGTGATAAC ATATGCACCT GCACAAAGAC**

**AACCTTGGCT GTGAGAGAGA TCCACTGCGC TCCGGGGGCA GTTAGCATCC**

**AAGGAGACTG CGCGTATGTT TCCAAGCCAT CGACCAGTTG CGAAGGGGTA**

**AGTACTTCAC TGCGTTTCGC TTTATCGTGT CTGAAAAAGA CGTCATTTCA**

**GGGACGGCTA CGAGGAGAAA AATGTATTCA GGATTTCATG GTGCCCCCCC**

**TCTGCGGATG A**

putative protein coding sequence in En_contig 2812

by joining of bp 2772..2831, 2886..4013;4067…4282

introns were confirmed by sequencing of cDNA

**MKYCMVAITLFCSVSGSRSLEAGDDYATTDGFTEELLRQLKVVPVPPAKC 50**

**QCQEGYDMRNGSCIRSAEFDPQDVCRSGKMVDGLCVTPAPQVLQCPHDYV 100**

**TVCKNKDRSESPCCAKEETAEKAARCRDGTDSHDGRCTRILARERVHECP 150**

**PGHALMSSHGTQCIKQEYGEAAPACVYPDELSPEGDSCLTTIQQGFEYIC 200**

**PDEYECVARSLKKKKKYSPLCSACVKIEKAQPTCGCPEGQDEFNGFCYEA 250**

**GTYEFCQSRKGLPQKQAPPRKKGYTAVELYEQQPEVNCKPLGHVTCTCDL 300**

**PFSLQGSGESSTCIHRDLIPAVPICRGQTDENGNCIAQVQKRVLYECAEG 350**

**FTCDVVNKKGRCNCVRLTAVEPTKRCASGEEREDKCIEIIEEPKILECPQ 400**

**GYSETCCDNICTCTKTTLAVREIHCAPGAVSIQGDCAYVSKPSTSCEGGR 450**

**LRGEKCIQDFMVPPLCG***

Deposited at GenBank: AJG00894.1


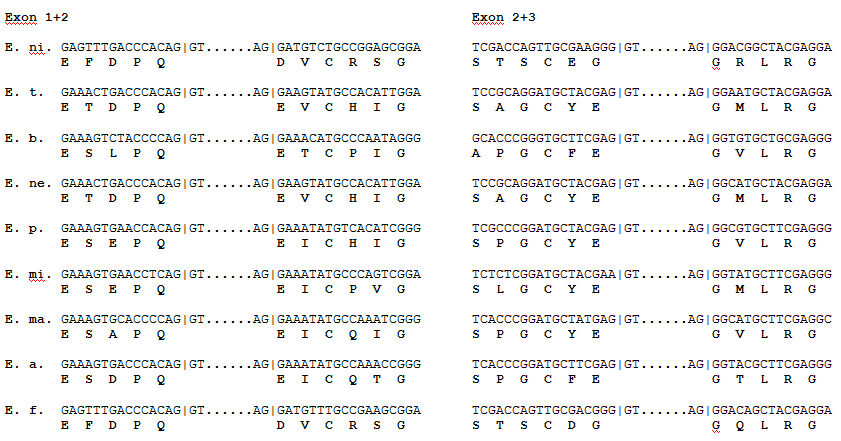
**B) Intron –exon structure of *Eimeria* OWP2 (1-9) listed in A)**

**C) BLAST results (E-value) of EnOWP2 and EnOWP6 vs. TgOWPs**

**(target: proteins; blastp)**

|  | EnCOWP2 (contig2812) | EnCOWP6 (contig6026) |
| --- | --- | --- |
| TgOWP1  TGVEG_204420  (*TGVEG_044810) | 4x10^-8^ | 1x10^-41^ |
| TgOWP2  TGVEG_209610  (*TGVEG_038950) | **10^-43^** | 2x10^-7^ |
| TgOWP3  TGVEG_268310  (*TGVEG_153310) | 4x10^-5^ | 9x10^-23^ |
| TgOWP4  TGVEG_222940  (*TGVEG_053360) | - | 0,96 |
| TgOWP5  TGVEG_248730  (*TGVEG_003060] | 4x10^-5^ | 5x10^-31^ |
| TgOWP6 TGVEG_286250  (*TGVEG_033590] | 2x10^-5^ | **1x10^-122^** |
| TgOWP7  TGVEG_210950  (*TGVEG_008810) | 10^-7^ | 4x10^-26^ |

***corresponding accession number denoted in Possenti *et al.* 2010**

**D)** **Clustal Omega Alignment of TgOWP2 (GenBank Accession No. EPT32301) and EnOWP2**

TgOWP2 MPTICSKIICALSVLLATTAHVPGMPALAS------TDATI--MKKAPGTYPAPPPDPTR

EnOWP2EnCOWP ------MKYCMVAITL--FCSVSGSRSLEAGDDYATTDGFTEELLRQLKVVPVP-----P

* ::: * . * * :* : **. : : . *.*

TgOWP2 ARCKCPFGFEKMDKSCVKKEAAGQPEAICQSGVLEDGKCRTRAAEAFRCPDGFETICDAN

EnOWP2 AKCQCQEGYDMRNGSCIRS-AEFDPQDVCRSGKMVDGLCVTPAPQVLQCPHDYVTVCKNK

*:*:* *:: : **::. * :*: :*:** : ** * * * :.::**. : *:*. :

TgOWP2 STAKSKCCRRTESQEINFKCAEGTTETIDGDCKRLKQFPPSHECPLGYRY---DERYCVR

EnOWP2 DRSESPCCAKEETAEKAARCRDGT-DSHDGRCTRILARERVHECPPGHALMSSHGTQCIK

. ::* ** : *: * :* :** :: ** *.*: **** *: . *::

TgOWP2 TEPGHVVPACGVESQLTA-HNSCLSIAPGEIVYECPVGFHCASN-----AKNSDFCKSCK

EnOWP2 QEYGEAAPACVYPDELSPEGDSCLTTIQQGFEYICPDEYECVARSLKKKKKYSPLCSACV

* *...*** .:*: :***: : * ** :.*.:. * * :*.:*

TgOWP2 RRELEPVSCECDAGTVESDGLCYQAEEYHECFDKIKKN--VVPTEVVDKDEDEKLDKKKD

EnOWP2 KIEKAQPTCGCPEGQDEFNGFCYEAGTYEFCQSRKGLPQKQAPPRKKGYTAVELYEQQPE

: * :* * * * :*:**:* *. * .: .* . * ::: :

TgOWP2 KKCE-TTRSKCSCRAGFNLVCKGKECHCVKEESAAVVRRCLGFDDGSGNCVRHLETAPVY

EnOWP2 VNCKPLGHVTCTCDLPFSLQGSGESSTCIHRDLIPAVPICRGQTDENGNCIAQVQKRVLY

:*: : .*:* *.* .*:.. *::.: .* * * * .***: :::. :*

TgOWP2 QCGEGQECEIVGKK-ECKCVYKIRKDSTINCGDGVLIGSDCFSVEHIPKTRHCQDGFDVA

EnOWP2 ECAEGFTCDVVNKKGRCNCVRLTAVEPTKRCASGEEREDKCIEIIEEPKILECPQGYSET

:*.** *::* ** .*:** : * .*..* ..*:.: . ** .* :*:. :

TgOWP2 CRRSECQCERNVFTRRVLTCDAEAAKKSEGCASLSEPEFICKEGQLINGNCVRLSYTVEL

EnOWP2 CCDNICTCTKTTLAVREIHCAPGAVSIQGDCAYVSKPSTSCEGGRLRGEKCIQDFMVPPL

* . * * :..:: * : * *.. . ** :*:*. *: *:* :*:: . *

TgOWP2 CEA

EnOWP2 CG-

*

**E) Analysis of orthologous EnOWP 2/6 ESTs in related Apicomplexa**

| **Name/ Database** | ***Eimeria* transcript database TDB) translated cDNA >100 blastp** | **Toxodb ESTs**  **tblastn** |
| --- | --- | --- |
| EnOWP2 | *Eimeria acervulina*  Eace_0293, complete,  E-value 10^-174^, stage: **Ou**, **pO**,**sO**  most counts in **Ou** | *Eimeria acervulina*  EST JK217832, E-value 2x10^-83^*,*  stage: **sporoblast-phase oocysts**  EST JK222052, E-value 2x10^-76^  stage: **unsporulated oocysts** |
|  | *Eimeria tenella*  Eten_1237, partial,  E-value 10^-152^ stage: **Ou**, **pO**,**sO**  most counts in **pO** | *Eimeria tenella*  EST AM264445, E-value 4x10^-84^  stage: **unsporulated oocyst** |
|  | *Eimeria maxima*  Emax_0264,complete,  E-value 10^-158^ , stage: **Ou**, **pO**,**sO**  most counts in **pO** | *Eimeria maxima*  EST JK234048, E- value 3x10^-64^  stage: **sporoblast-phase oocysts** |
|  |  | *Toxoplasma gondii*  inter alia EST BU790677, E-value 2x10^-11^  stage: **partially sporulated oocysts** |
| EnOWP6 | *Eimeria acervulina*  Eace_0415, partial 5x10^-74^  stage:unknown*  Eace_0779, partial 4x10^-68^  stage: **Ou** | *Eimeria acervulina*  EST JK224778 E-value 10^-54^  stage: **unsporulated oocysts** |
|  | *Eimeria tenella*  No hit (result E-values >10^-10^) | *Eimeria tenella*  No Hit (result E-values >10^-10^) |
|  | *Eimeria maxima*  Emax_0113, partial 6x10^-43^  Stage: **Ou** | *Eimeria maxima*  EST JK248473 , E-value 2x10^-38^  stage: **unsporulated oocyst** |
|  |  | *Toxoplasma gondii*  EST BM131489 E-value 4x10^-08^  stage: **partially sporulated oocysts** |

*TDB covers unsporulated oocysts (Ou), sporoblast-phase oocysts (pO), sporulated oocysts (sO), sporozoites (s) and second generation merozoites stages (Mz1 and Mz2).

**F**) **EnOWP6**

*Eimeria nieschulzi* EnOWP6-locus (inverted) with intron and exons in contig Enie_6026 (GenBank ASM82694v1)

**ATGGGACACT TGGCTGTTTC TCTCCAAAGG GGAACCTGTT ATGTGTCGGT**

**GTTGCTCCTG CTCCTCTCTT TTTCCGTAAG AGAAGTCGCA GCAAGAGGAG**

**ATGAAGACGT TATGCAGGCT GCTGAGGAAT GCCATCACGG CTGGACGCTC**

**ACCAACGGCG AGTGTGTAAA GGAGGTTGCC CGCAAGTCTC TCAGCCCCTG**

**TCCCCCCAAG GCGTCATTAG AGGTGAGTTT GGCTTTCCTC TGCTTCCCGT**

**TGCGTGCCCC TAATGAATTT TTTAAAGCAG GTAAAACTGT GCATGCATGG**

**GTCTGATGGT AGCCACTGGA AGTTGTCCGT TTTGCGTCTC TCCAGAACGG**

**CCAGTGCGTC ATCGAGAGAG CCACGAAGGC TTCCCTGGTG TGTGCCCCCG**

**GGCAAAAGTT GGTCGGCGAA AAATGTGAAT ATGAGGAGAA GACGGTGGCT**

**ATCTCTTCGT GTCCTAAGGG GTTTGTATTC AACGGGGTGG GTTGCAGCAA**

**GAGCCAAGAG GTTGTGGGAA TGGTCGCCTG TCCCCACGGC TACAAGGCGA**

**ACGAGAGCAG AGAGCTTTGT GTTCGGGAAA TAAAACACAA GGCCTCAACG**

**TTCTGTCCAC CGGGATCTCA GGCGTACGGA CAGGACAAGT GCCTAATGAC**

**AGACAATTTG CCGGTGGTAA GGACGTGTGG GCTTGGCTAC ATGCTGGTCG**

**ACGGCAACTG CGTGAAGCAA GATTACACCC GAGCAGAGCT GCAGTGTCCC**

**ACAGGATACA AGCTGGATGA GGGAGTGTGT CGCTCTTCCC AGAAAGTCCC**

**AGCTAATGCG GTGTGTCCTG CTTCTCACGA GTTCGACGGA AGAGAGTGCA**

**TTAAAGTAGT GCTGGTAGAT CCAACTCCCT CTTGCCACGC TGGCTATCAC**

**GTCAGCGGCA ACAAGTGCCT CAAGGTCTTG GAAAAGGACG TCATTCTTTA**

**CTGCCCCGTG GGTTTCGAGC TTTTGGAAGA CAAGTGCGTC AGCAGGACAG**

**CCGTGGCAGC CAAGAGTGAA TGTCCCGAGG GCACTCTCAG CACCACCATG**

**CTGGGTAAAA GCGTATGTGA GACCATCTCA GCCAACCCTC CCGCTCTGCG**

**CTGTCACGGA AATCAAGTCC TCGAGGGCGA CCGCTGCGTT AAACGGACCA**

**CTGGGCACGT GAAGACCGGG TGCCCCTTGG GCTACAAGCT CTCAGGCGAT**

**GGCTGCAGCA AGGAGCACTT CTTAGCTCCG TCACAGCTCT GTGCTGATGG**

**ATCTACTCTC AAAGACGGAT ACTGCCTGCA GGTGGAAACC ATTCCCAGCA**

**AGCCGCACTG CCTGGATGGA GAGCCCACGC CTCAGGGAAC TTGCGTGCGA**

**CTCTATGCAG CAGATTTGAT ATATAATTGT CCTTTGGGCT TCAAGCTGGT**

**CGGGTCTAAC TGCAGCAGGC TCTCTCACAC GCATGCAGCC TCTGTCTGCC**

**CCTTAGAAAC GAAGATGAGG GGAGGGGAGT GTATTGTGAC GGAGACAGTT**

**GCCTCCAATC CGCAATGTGA AGAGCCTTTT CAACCAGATG CAGCAGGGGG**

**ATGTGTAAAG CTGGAGTACA AACCGGCGAA ACGCACCTGC CCAAAGACTC**

**ACAAACTATT CAATAATTAC TGCGTCAGGA AGGCGGGACA CGCGCTCTTC**

**AGGGGAGAAC CCATCTAA**

putative protein coding sequence in contig Enie_6026

by joining of bp 5977...6198, 6322…7644

intron confirmed by sequencing of CDNA

MGHLAVSLQRGTCYVSVLLLLLSFSVREVAARGDEDVMQAAEECHHGWTL 50

TNGECVKEVARKSLSPCPPKASLENGQCVIERATKASLVCAPGQKLVGEK 100

CEYEEKTVAISSCPKGFVFNGVGCSKSQEVVGMVACPHGYKANESRELCV 150

REIKHKASTFCPPGSQAYGQDKCLMTDNLPVVRTCGLGYMLVDGNCVKQD 200

YTRAELQCPTGYKLDEGVCRSSQKVPANAVCPASHEFDGRECIKVVLVDP 250

TPSCHAGYHVSGNKCLKVLEKDVILYCPVGFELLEDKCVSRTAVAAKSEC 300

PEGTLSTTMLGKSVCETISANPPALRCHGNQVLEGDRCVKRTTGHVKTGC 350

PLGYKLSGDGCSKEHFLAPSQLCADGSTLKDGYCLQVETIPSKPHCLDGE 400

PTPQGTCVRLYAADLIYNCPLGFKLVGSNCSRLSHTHAASVCPLETKMRG 450

GECIVTETVASNPQCEEPFQPDAAGGCVKLEYKPAKRTCPKTHKLFNNYC 500

VRKAGHALFRGEPI*

**G)** Clustal Omega Alignment of TgOWP6 (ToxodbID TGVEG_286250) and EnOWP6

TgOWP6 MAPSRRRSKLVSLFAAGLCLVAPHAWNAGAQEIVPLIAGNEDVAAALEECPTGYNLDGGV

EnOWP6 ------MGHLAVSLQRGTCYVSVLLLLLSFSVREVAARGDEDVMQAAEECHHGWTLTNGE

.:*. : * * *: . . *:*** * *** *:.* *

TgOWP6 CKKEIARRPMTLCPPRASFEGGECVTEKEVKSVLTCGADELLVGDRCEVEEFIKALASCP

EnOWP6 CVKEVARKSLSPCPPKASLENGQCVIERATKASLVCAPGQKLVGEKCEYEEKTVAISSCP

* **:**: :: ***:**:* *:** *: .*: *.*. : ***::** ** *::***

TgOWP6 RDYTFTGAACMRSQESKAQQYCDDGYKLS-SGDLCLRDIKEKPEAVCPPGARQQG-DKCF

EnOWP6 KGFVFNGVGCSKSQEVVGMVACPHGYKANESRELCVREIKHKASTFCPPGSQAYGQDKCL

: :.*.*..* :*** . * .*** . * :**:*:**.* .:.****:: * ***:

TgOWP6 ILESSPPHVACTRGFELESGMCVRVETVRPEQRCPHGYRLDGGICRNVVRLQPNAVCPAG

EnOWP6 MTDNLPVVRTCGLGYMLVDGNCVKQDYTRAELQCPTGYKLDEGVCRSSQKVPANAVCPAS

: :. * :* *: * .* **: : .* * :** **:** *:**. :: ******.

TgOWP6 HDFNGKECVMSQLAEPTLKCDDGYQLEGTVCVKRLEKAAKPECPAKYEYRNNVCIKQTAV

EnOWP6 HEFDGRECIKVVLVDPTPSCHAGYHVSGNKCLKVLEKDVILYCPVGFELLEDKCVSRTAV

*:*:*:**: *.:** .*. **::.*. *:* *** . **. :* :: *:.:***

TgOWP6 KPRSECPEGSVEAAGPGK-TCEAVHIADSSLVCPNDFSIYNGQCVRKTTGPMHQECALGF

EnOWP6 AAKSECPEGTLSTTMLGKSVCETISANPPALRCHGNQVLEGDRCVKRTTGHVKTGCPLGY

:******::.:: ** .**:: :* * : : :**::*** :: * **:

TgOWP6 RNTREGVCVRETHQKAEKRCPEGLELSGDGVCTAQDSAPAHLVCDQGELTPQGTCVRVFT

EnOWP6 K-LSGDGCSKEHFLAPSQLCADGSTL-KDGYCLQVETIPSKPHCLDGEPTPQGTCVRLYA

: : :* :* . .: * :* * ** * :: *:: * :** ********:::

TgOWP6 AETLFTCPRGFKLREPDCIRQVQKAAAATCPENAKMRGGNCIVLERFPASDNCEDDYVSD

EnOWP6 ADLIYNCPLGFKLVGSNCSRLSHTHAASVCPLETKMRGGECIVTETVASNPQCEEPFQPD

*: ::.** **** :* * :. **:.** ::*****:*** * . :. :**: : *

TgOWP6 GHSSCIKYDIKSPKRTCPRAYRLFNTVCVKRLFRL-------

EnOWP6 AAGGCVKLEYKPAKRTCPKTHKLFNNYCVRKAGHALFRGEPI

. ..*:* : * *****::::***. **:: :

H) Clustal Omega Alignment EnOWP6 with ToxoDB entries of other *Eimeria* species

EnOWP6 MGH-LAVSLQRGTC-----------------------------YVSVLLLLLSFSVREVA

EfaB_MINUS_1001.g115 MGR-LAVSLQRGAC-----------------------------YVSMLLLVLSFSTREVA

ETH_00012470 MGLSLRVGADASACAAAPAAAPAAA--AAAAAGPPRGRRRGAFLLSGLLLLCVFAPTPAA

ENH_00025410 MGLSLRVGAAASACAAAPAAAPAAAAAAAAAAGPPRGRRRGAFLLSGLLLLCVFAPTPAA

** * * * . . :**: :* ***: *: .*

EnOWP6 ARGDEDVMQAAEECHHGWTLTNGECVKEVARKSLSPCPPKASLENGQCVIERATKASLVC

EfaB_MINUS_1001.g115 ARGDEDVMEAAEECHHGWTLTNGECVKEVARKSLSPCPPKASLENGQCVIERATKSSLVC

ETH_00012470 ALGDEDVMDAAEECHAGWTLLNGQCIKEKERKPISPCPPKAALDGAECVLERASKAELSC

ENH_00025410 ALGDEDVMDAAEECHAGWTLLNGQCIKEKERKPISPCPPKAALDGAECVVERATKAELSC

* ******:****** **** **:*:** ** :*******:*: .:**:***:*:.* *

EnOWP6 APGQKLVGEKCEYEEKTVAISSCPKGFVFNGVGCSKSQEVVGMVACPHGYKANESRELCV

EfaB_MINUS_1001.g115 APGQKLIGEKCEYEEKTVAISSCPKGFVFNGVGCSKSQEVVGMVSCPHGYKANESRELCV

ETH_00012470 AAGQQLVGEKCEFEEKTTAISSCPKHFIFNGVSCSKTQEVAAGVSCPPGFKPSEDREVCF

ENH_00025410 AVGQQLVGEKCEFEEKTTALSSCPKNFVFNGVSCSKTQEVAAAVSCPPGFKPSEDREVCF

* **:*:*****:****.*:***** *:****.***:***.. *:** *:* .*.**:*.

EnOWP6 REIKHKASTFCPPGSQ---AYGQDKCLMTDNLPVVRTCGLGYMLVDGNCVKQDYTRAELQ

EfaB_MINUS_1001.g115 REIKHKASTLCPPGSQ---AYGQDKCLMTDNLPASRNCGLGFVLTDGNCIKQEYTRAELQ

ETH_00012470 RESKQKAAAVCPPGAVLQQHGGHEKCLVEETVAAAQSCPLGFSLQNGSCSKLEVSPPQQQ

ENH_00025410 RESKQKAAAVCPPGAVLQQQGGHEKCLVEETAAAAQSCPLGFVLQNGSCSKLEVSPPQQQ

** *:**::.****: *::***: :. . :.* **: * :*.* * : : : *

EnOWP6 CPTGYKLDEGVCRSSQKVPANAVCPASHEFDGRECIKVVLVDPTPSCHAGYHVSGNKCLK

EfaB_MINUS_1001.g115 CPTGFKLDEGVCRSTQKVPANAVCPADHEFDGRECIKVVLADPTPSCHAGYHVSGNKCLK

ETH_00012470 CPLGAVLQEGLCRSSQRVAANPVCPPDFEFDGKECLLLLLSPPTPSCPPGYHLGDAKCLK

ENH_00025410 CPLGALLQDGLCRSSQRVAANPVCPPDFEFDGKDCLLLLLSAPSSSCPPGYHLAENKCLK

** * *::*:***:*:* ** *** ..****::*: ::* *: ** ***:. ****

EnOWP6 VLEKDVILYCPVGFELLEDKCVSRTAVAAKSECPEGTLSTTM------------LGKSVC

EfaB_MINUS_1001.g115 VLEKDVILYCPVGFELIDDKCVSRTSVAAKSECPQGTLSTTL------------LGKSIC

ETH_00012470 VLEKELLLLCPKDSELLEGQCVQRRTAKASSVCPEGSRSSGKGAAAAAGGALLQRSSSSC

ENH_00025410 VLEKELLLLCPKDSELLEGQCVQRRTAKASSVCPEGSRSSGKAA-AAAGGPLLQRSSSSC

****:::* ** **:: :**.* :. *.* **:*: *: ..* *

EnOWP6 ETISANPPALRCHGNQVLEGDRCVKRTT-GHVKTGCPLGYKLSGDGCSKEHFLAPSQLCA

EfaB_MINUS_1001.g115 ETISANPPALRCHGSQVLEGDRCVKRIT-GHVKTGCPLGYKLSGDGCTKEHFLPPTHLCA

ETH_00012470 EVVSSSPPALKCPPEALLEAGQCVRRAAAAPLGPSCPLGFRSSGQGCSKEALVGPQLLCP

ENH_00025410 EVLSSSPPALKCPPDALLEAGQCVRRAAAAPLGPSCPLGFRSSGQGCSREALVGPQLLCP

*.:*:.****:* . :**. :**:* : . : .****:: **:**::* :: * **

EnOWP6 DGSTLKDGYCLQVETIPSKPHCLDGEPTPQGTCVRLYAADLIYNCPLGFKLVG-------

EfaB_MINUS_1001.g115 DGSNLKDGYCVQVETIPSKPHCLDGEATPQGTCVRLYAADLIYNCPLGFKLVG-------

ETH_00012470 QGAPLKDRFCVSTETAAARPFCPEGEATPQGTCVRLFEAPLLLNCPMGFKLQQGSS----

ENH_00025410 QGALLKDKYCVSTETAAAKPFCPEGEATPQGTCVRLFEAPLLLNCPMGFKLQQGSSSSSS

:*: *** :*:..** ::*.* :** *********: * *: ***:****

EnOWP6 -SNCSRLSHTHAASVCPLETKMRGGECIVTETVASNPQCEEPFQPDAAGGCVKLEYKPAK

EfaB_MINUS_1001.g115 -SSCSRLTHTHAASVCPLDTKMRGGECIVTETVSSNPQCEEPFQPDAQGGCVKLEYKPAK

ETH_00012470 --SCSRKSHAHAACTCPLNAKLRGEECIWTERIPANDSCEEGFQKDNKGFCLKLESKPPK

ENH_00025410 SSSCSRKSLAQAACTCPLNAKLRGQDCVWTERLPANDNCEEGFQKDGKGFCLKLEAKPPK

.*** : ::**..***::*:** :*: ** : :* .*** ** * * *:*** ** *

EnOWP6 RTCPKTHKLFNNYCVRKAGHALFRGEPI

EfaB_MINUS_1001.g115 RTCPKTHKLFNNYCVRKAGHALFRGEPL

ETH_00012470 RSCPKTHKLFNTYCVRKVPGALFRGHPL

ENH_00025410 RSCPKTHKLFNTYCVRKVPGALFRGHPL

*:*********.*****. *****.*:
