# Supplementary figures and images for: Two COWP-like cysteine rich proteins from Eimeria nieschulzi (coccidia, apicomplexa) are expressed during sporulation and involved in the sporocyst wall formation
Source: Parasit Vectors. 2015 Jul 25;8:395. doi: 10.1186/s13071-015-0982-3 (PMC4514997; doi:10.1186/s13071-015-0982-3)

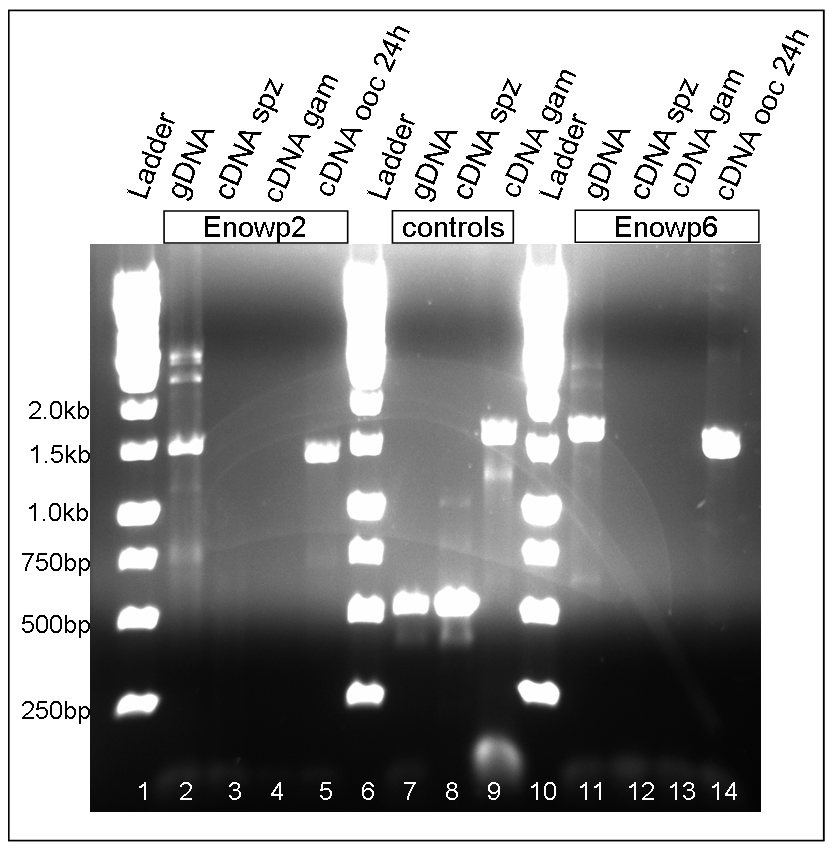

Supplement: Additional file 3: Figure S2. — Stage specific expression EnOWP2 and EnOWP6. Transcripts of EnOWP2 (Lane 5) and EnOWP6 (Lane 14) had been amplified from cDNA of sporulating oocyst (24 h), but not from sporozoite cDNA (Lane 3 and 12) or cDNA derived from gamonts (Lane 4 and 13). From genomic DNA both intron harbouring genes had been amplified (Lane 2 and Lane 11), which resulted in longer amplification products than from cDNA. To ensure gamont cDNA quality the gametocyte specific gene EnGam82 had been utilized and was successfully amplified von gamont cDNA (Lane 9). Further small ribosomal subunit gene was successfully amplified from genomic DNA and sporozoite cDNA which reveals sufficient template quality. [file 13071_2015_982_MOESM3_ESM.tif]

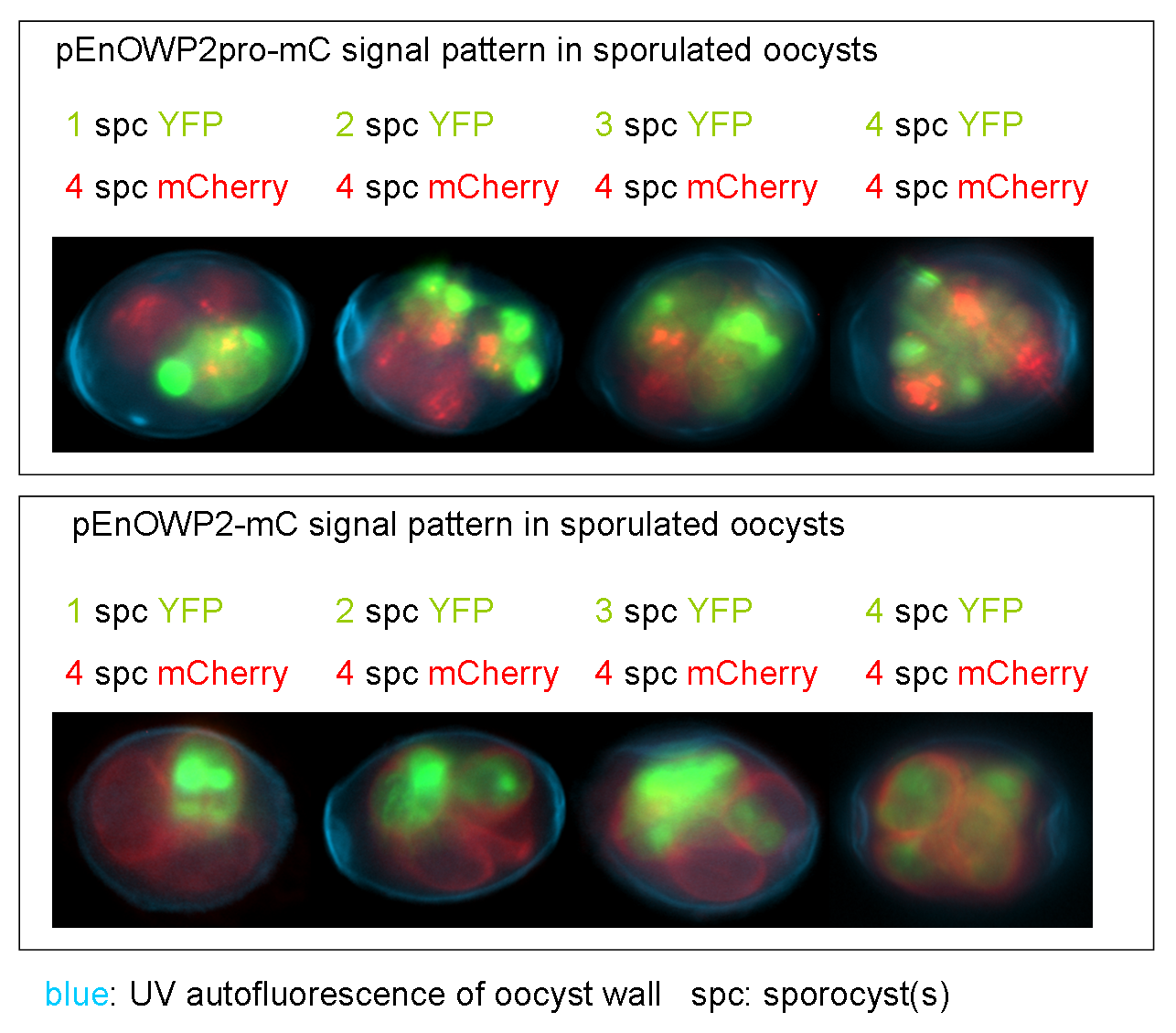

Supplement: Additional file 4: Figure S1. — Distribution of YFP and mCherry signal in pEnOWP2Pro-mC and pEnOWP2Pro-mC. The different distribution of fluorescence signal within these transgenic lines is visualized in this figure. The YFP signal alters in sporulated oocysts and one, two, three, or four sporocysts, harbouring YFP expressing sporozoites, were observed in the single oocysts. The mCherry signals were distributed to all sporocyst and did not alter. See also Additional file 5 and Additional file 6. For quantifications see Table 1. [file 13071_2015_982_MOESM4_ESM.tif]

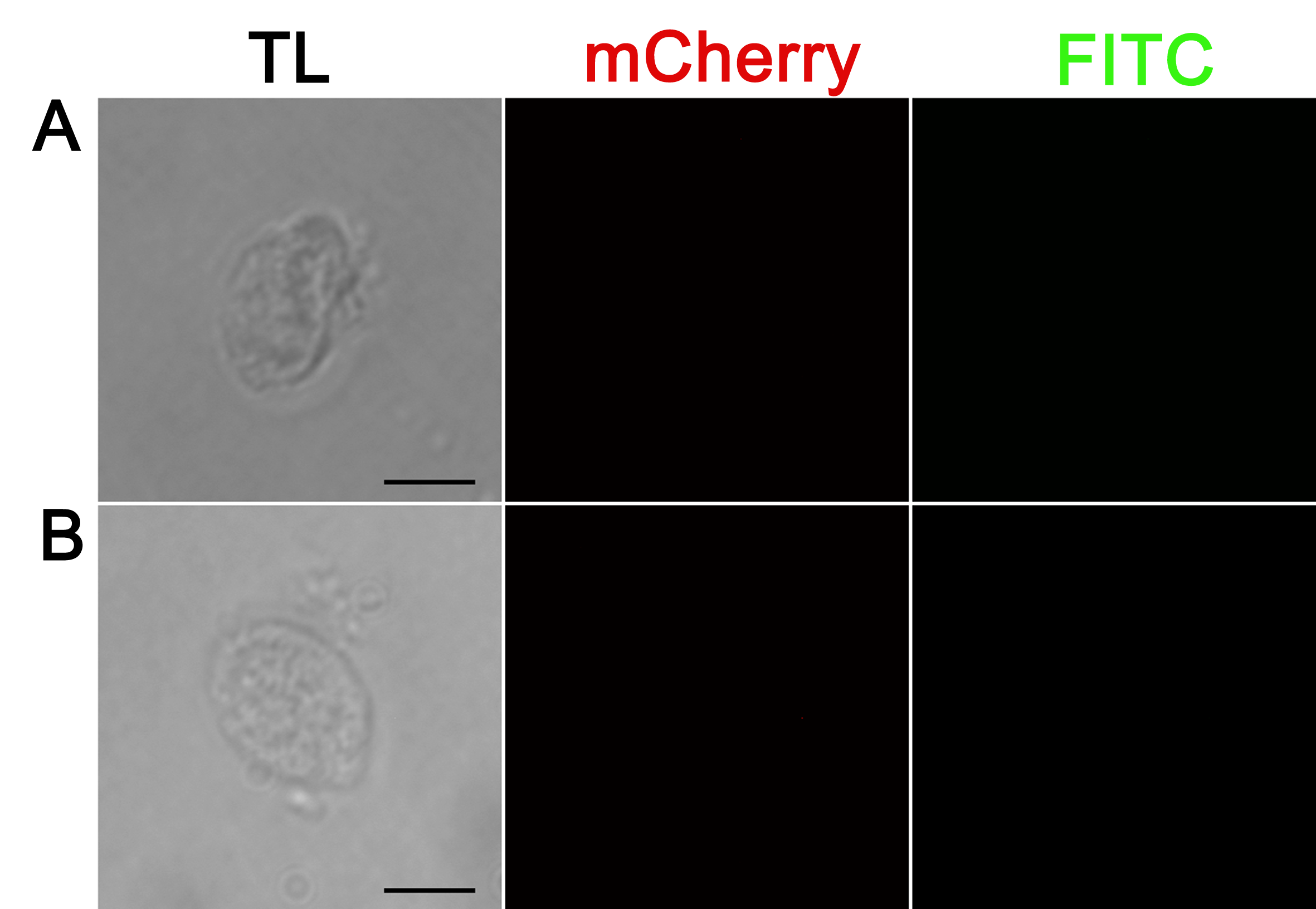

Supplement: Additional file 7: Figure S3. — IFA with anti-mCherry antibody on wild type sporocysts. Excysted wild type sporocyst were MeOH fixed and permeabilised triton x-100 (A) or used unfixed and not permeabilised (B) as negative control for anti-mCherry antibody test. Neither the mCherry nor the FITC channel fluorescent signals were detectable independently of the fixation or permeabilisation. Scale bar 5 μm. TL: Transmitted light; mCherry: mCherry fluorescence channel, FITC: FITC fluorescence channel. [file 13071_2015_982_MOESM7_ESM.tif]

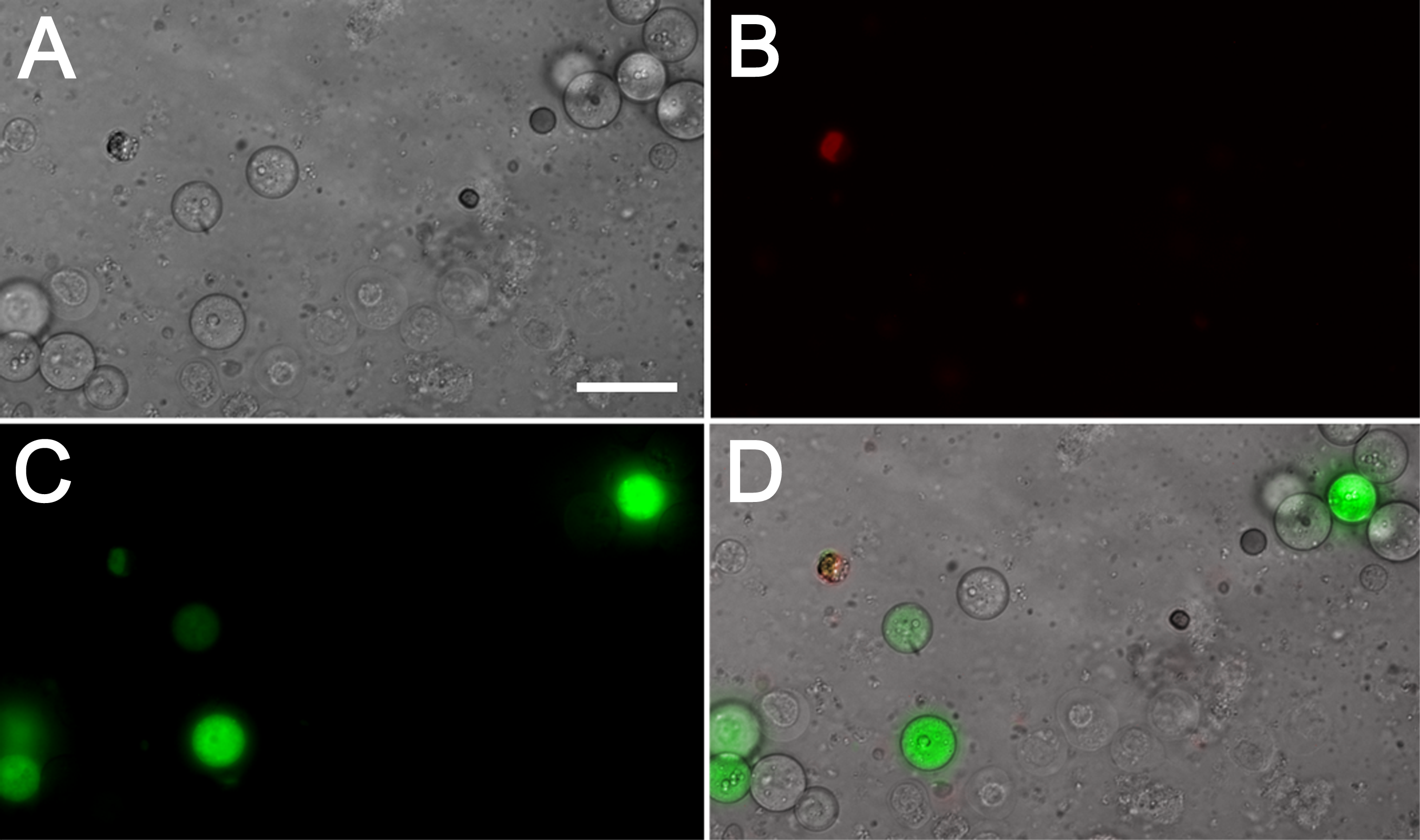

Supplement: Additional file 8: Figure S4. — Gamonts of pEnOWP6mc transformed parasites (P1). At 172 h p.i. we could observe non fluorescent, and only YFP expressing macrogamonts, without mCherry signal indicating absence of EnOWP6 in the gamont stage. Scale bar 20 μm. A: Transmitted light B: mCherry channel C: YFP channel. D: Overlay of A,B and C. [file 13071_2015_982_MOESM8_ESM.tif]
